# The African swine fever virus MGF360-16R protein functions as a mitochondrial-dependent apoptosis inducer by competing with BAX to bind to the HSP60 protein

**DOI:** 10.1128/jvi.01401-24

**Published:** 2025-02-27

**Authors:** Zhiyong Xiang, Zhen Xu, Wenlian Weng, Hua Wang, Jiajun Wu, Fei Jiang, Yajin Qu, Quanlin Li, Peng Gao, Lei Zhou, Xinna Ge, Xin Guo, Jun Han, Yongning Zhang, Hanchun Yang

**Affiliations:** 1 National Key Laboratory of Veterinary Public Health and Safety, College of Veterinary Medicine, China Agricultural University 630101 , Beijing, China; 2 Key Laboratory of Animal Epidemiology of Ministry of Agriculture and Rural Affairs, College of Veterinary Medicine, China Agricultural University, Beijing, China; 3 China Animal Disease Control Center, Beijing, China; Northwestern University Feinberg School of Medicine, Chicago, Illinois, USA

**Keywords:** African swine fever virus (ASFV), apoptosis, BAX, HSP60, MGF360-16R, porcine macrophages

## Abstract

Induction of cell apoptosis is a critical pathogenic feature of the African swine fever virus (ASFV), a devastating threat to the world pig industry, but the underlying mechanisms have remained unclear. Here, we report the genome-wide screening and identification of 27 ASFV-encoded apoptosis inducers. Of them, the viral protein MGF360-16R was found to be a mitochondria-targeting protein and exhibits strong activity to induce mitochondrial-dependent apoptosis. Interestingly, this protein exhibits a dynamic distribution pattern during viral infection of porcine macrophages; it is mainly localized to the viral factory in the early stage and then shifts a portion to mitochondria in the late stage, a timing that coincides with virus-induced apoptosis. Mechanistically, MGF360-16R interacts with and competes for the cellular heat shock protein 60 (HSP60), and this results in the release of BAX from the HSP60-BAX complex, leading to the activation of BAX and induction of apoptosis. The ASFV mutant lacking MGF360-16R showed a reduced ability to induce cellular apoptosis, highlighting the complex nature of ASFV-induced cell apoptosis. Overall, our study adds a new function to MGF360-16R and reveals a novel mechanism of ASFV-mediated cell apoptosis.

**IMPORTANCE:**

ASFV is a complex virus with a huge genome and numerous encoded proteins. Induction of cell apoptosis is associated with pathological damage in pigs caused by ASFV, but its inducers and underlying mechanisms remain unclear. Through genome-wide screening, the viral protein MGF360-16R was identified as a potent apoptosis inducer with a unique localization transfer from the viral factory to mitochondria during ASFV infection of porcine macrophages. MGF360-16R induces apoptosis by interacting with the cellular HSP60 protein to release BAX from the HSP60-BAX complex in a competitive binding manner. Our research findings not only reveal a novel function of MGF360-16R but also provide clues for understanding the pathogenesis of ASFV and potentially developing new therapeutic strategies.

## INTRODUCTION

African swine fever virus (ASFV) is a large and structurally complex virus that usually causes a highly lethal disease called African swine fever (ASF) in domestic pigs and Eurasian wild boars (1). The virus has a linear double-stranded DNA genome of about 170‒193 kbp, encoding approximately 150‒200 proteins (2). However, the function of most ASFV proteins is still unclear. Taxonomically, ASFV is classified in the genus *Asfivirus* within the family *Asfarviridae* (3). Clinically, ASFV infection mainly causes gross pathological lesions such as extensive hemorrhage of skin and various internal organs, including but not limited to the spleen, lymph nodes, thymus, and tonsils (4). The microscopic pathological lesions mainly include the destruction of porcine monocytes and macrophages, depletion of lymphocytes, and death of infiltrating lymphocytes, which have been attributed at least in part to the massive cell apoptosis induced by ASFV infection (5–7).

ASFV has a strong tropism for the mononuclear phagocyte system, especially monocytes and macrophages (8). *In vivo* infection experiments in pigs showed that ASFV can not only induce apoptosis of target cells (7, 9) but also induce apoptosis of bystander cells such as thymocytes and lymphocytes (5, 10). *In vitro* infection experiments showed that ASFV can induce apoptosis of both primary and passage cells, such as primary porcine macrophages and Vero cells (11, 12). In the case of virus-infected cells, induction of early apoptosis will severely restrict the production of progeny viruses and reduce or eliminate their spread in the host. Therefore, many viruses have evolved various strategies to evade or delay the early onset of apoptosis to allow the production of high-yield progeny viruses. However, apoptosis also represents a very potent mechanism by which the virus can induce cell death, facilitating progeny virus dissemination and immune escape while limiting the induction of inflammation and immune response (13). Our recent study showed that ASFV can induce the formation of apoptotic bodies in primary porcine alveolar macrophages (PAMs) in the late stage of infection, enabling the virus to achieve effective intercellular transmission and invasion of naive cells, while escaping neutralization by swine serum antibodies (14).

Currently, the studies on ASFV-induced apoptosis have mainly focused on identifying its apoptosis inducers one by one and elucidating their molecular mechanisms of action. To date, only three ASFV proteins have been reported to induce apoptosis, including E183L (p54), E199L, and EP402R (CD2v) (12, 15, 16). There has been no report on the systematic screening of ASFV-encoded apoptosis inducers at the genome scale. Here, we performed the first genome-wide screening of ASFV proteins capable of inducing apoptosis, with the aid of immunoblotting to detect caspase-3 (CASP3) activation and confocal immunofluorescence to analyze the co-localization of ASFV proteins and mitochondria. The viral protein MGF360-16R, which displayed the most pronounced co-localization with mitochondria and strong activation of CASP3, was selected to investigate its molecular mechanism of inducing apoptosis.

## RESULTS

### Genome-wide screening of ASFV proteins with apoptosis-inducing potential

**Fig 1:**
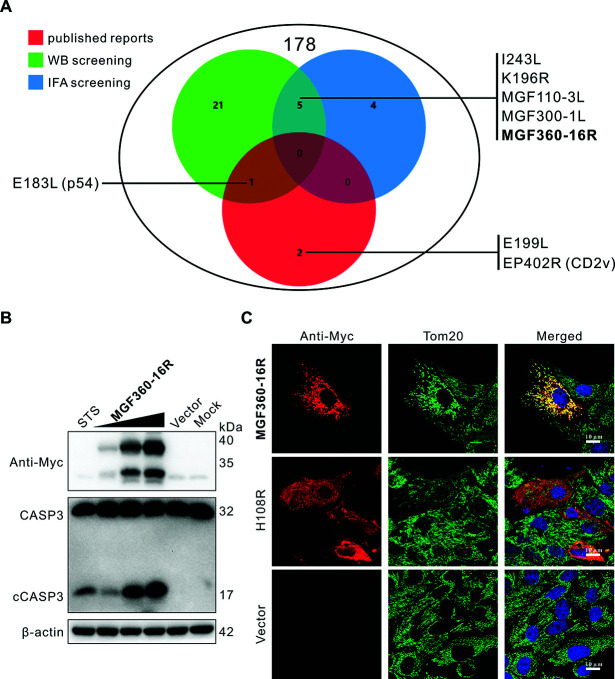
Genome-wide screening of ASFV proteins with the potential to induce apoptosis. (**A**) A total of 178 ASFV proteins were subjected to immunoblotting detection of CASP3 activation and confocal immunofluorescence analysis of co-localization with mitochondria in transfected WSL. Of the 178 ASFV proteins tested, 27 proteins were found to activate CASP3, 9 proteins were found to co-localize with mitochondria, and 5 proteins were found to both activate CASP3 and co-localize with mitochondria. The three previously reported apoptosis-inducing proteins, E183L (P54), E199L, and EP402R (CD2v), were included in the 178 proteins, but only E183L was proved to activate CASP3 as identified by our screening system. The detailed information of all proteins is summarized in Table S1 and Fig. S1 and S2, (**B**) WSL was transfected with either an empty vector (pcDNA3.1) or 1, 2, and 3 µg of a recombinant plasmid expressing Myc-tagged MGF360-16R protein. Cells were harvested after 42 h for immunoblotting using antibodies against Myc, CASP3, and β-actin. Cells treated with 2 µM of STS for 4 h were used as a positive control for apoptosis activation. Representative results from experiments performed in three independent biological replicates are shown. (**C**) WSL was transfected with either pcDNA3.1 or recombinant plasmids expressing Myc-tagged MGF360-16R or H108R proteins for 24 h. The cells were then analyzed by confocal immunofluorescence using mouse anti-Myc mAb and rabbit anti-Tom20 pAb as the primary antibodies, followed by immunostaining with Alexa Fluor 488-conjugated goat anti-rabbit and Alexa Fluor 568-conjugated goat anti-mouse IgG secondary antibodies.

To screen ASFV-encoded apoptosis inducers at the genome scale, immunoblotting was used to detect CASP3 activation in wild boar lung cells (WSL) ectopically expressing ASFV proteins. Staurosporine (STS)-treated WSL was used as a positive control for apoptosis. With the aid of this screening method, a total of 27 proteins capable of activating CASP3 were screened from the 178 ASFV proteins that could be ectopically expressed in WSL (Fig. 1A; Table S1). The immunoblotting screening results of these 27 proteins are shown in Fig. 1B and Fig. S1, all of which activated CASP3 in a dose-dependent manner, producing a cleaved form of CASP3 (cCASP3) similar to the STS control. Among them, the MGF360-16R protein exhibited the strongest CASP3 activation phenotype (Fig. 1B; Fig. S1). By contrast, A238L, one of the representative proteins judged as negative, was not found to activate CASP3 through immunoblotting screening, as shown in Fig. S1 (in blue boxes). In addition, subcellular localization analysis of these 178 proteins in transfected WSL by confocal immunofluorescence assay showed that a total of 9 ASFV proteins could co-localize with mitochondria (Fig. 1A; Table S1). Among them, MGF360-16R showed the most pronounced co-localization with mitochondria (Fig. 1C; Fig. S2). In contrast, the confocal immunofluorescence results of H108R, one of the representative proteins of ASFV that didn't co-localize with mitochondria, are shown in Fig. 1C. Notably, a total of five proteins (I243L, K196R, MGF110-3L, MGF300-1L, and MGF360-16R) were screened out in both methods (Fig. 1A; Table S1). Of the three previously reported ASFV-encoded apoptosis inducers p54, E199L, and CD2v (12, 15, 16), p54 protein (E183L) was also screened out by immunoblotting (Fig. S1), confirming the reliability of our screening system. Since MGF360-16R can strongly activate CASP3 and obviously co-localize with mitochondria, we selected this protein for subsequent functional studies.

### MGF360-16R protein is localized to mitochondria and activates CASP3 through its 1‒145 amino acids (aa) domain

**Fig 2:**
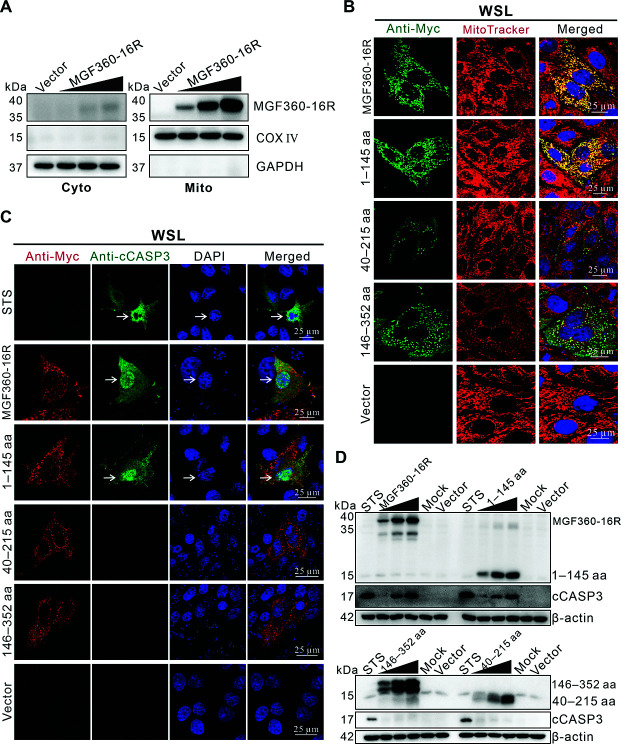
MGF360-16R protein is localized to mitochondria and activates CASP3 through its 1‒145 amino acids domain. (**A**) WSL was transfected with either empty vector (pcDNA3.1) or 10, 20, and 30 µg of recombinant plasmid expressing Myc-tagged MGF360-16R for 36 h. The cytosolic (Cyto) and mitochondrial (Mito) fractions of the cells were separated and detected by immunoblotting using antibodies against MGF360-16R, COX IV, and GAPDH. Representative results from experiments performed in three independent biological replicates are shown. (**B**) WSL was transfected with either pcDNA3.1 or each of its recombinant plasmids expressing Myc-tagged MGF360-16R, truncations 1–145 aa, 40–215 aa, and 146–352 aa, respectively, for 24 h. The cells were first stained with MitoTracker Red CMXRos 568 and then analyzed by confocal immunofluorescence using mouse anti-Myc mAb as the primary antibody. (**C**) WSL was transfected as described in (**B**). The cells were analyzed by confocal immunofluorescence using mouse anti-Myc mAb and rabbit anti-cCASP3 mAb as the primary antibodies. Cells treated with 2 µM of STS for 4 h were used as a positive control for apoptosis activation. (**D**) WSL was transfected with either pcDNA3.1 or 1, 2, and 3 µg of recombinant plasmids expressing Myc-tagged MGF360-16R or truncations 1–145 aa, 40–215 aa, and 146–352 aa, respectively, for 36 h. The cells were detected by immunoblotting using antibodies against Myc, CASP3, and β-actin. Representative results from experiments performed in three independent biological replicates are shown.

Recombinant plasmids expressing MGF360-16R at low, medium, and high doses were transfected into WSL, and its mitochondrial and cytosolic fractions were separated and detected by immunoblotting. Figure 2A shows that MGF360-16R was mainly distributed in the mitochondrial fraction in a dose-dependent manner and was almost undetectable in the cytosolic fraction, confirming that MGF360-16R was localized to mitochondria. Next, to identify the mitochondrial localization signal of MGF360-16R, three overlapping truncations (1–145 aa, 40–215 aa, and 146–352 aa) were constructed and transfected into WSL for confocal immunofluorescence analysis. Of them, only 1–145 aa still co-localized well with the mitochondrial staining signals, similar to the full-length MGF360-16R (Fig. 2B), suggesting that the 1–145 aa is the functional domain of MGF360-16R localized to mitochondria. Confocal immunofluorescence analyses using a monoclonal antibody (mAb) that could specifically recognize the cleaved (active) form of CASP3 (cCASP3) further showed that only truncation 1–145 aa was able to cause CASP3 entry into the nucleus, as the full-length MGF360-16R did (Fig. 2C), indicating that the 1–145 aa also served as the functional domain of MGF360-16R activating CASP3. Furthermore, immunoblotting results further showed that, similar to the full-length MGF360-16R, only truncation 1–145 aa activated CASP3 in a dose-dependent manner (Fig. 2D).

### MGF360-16R protein induces mitochondrial-dependent apoptosis by activating BAX

**Fig 3:**
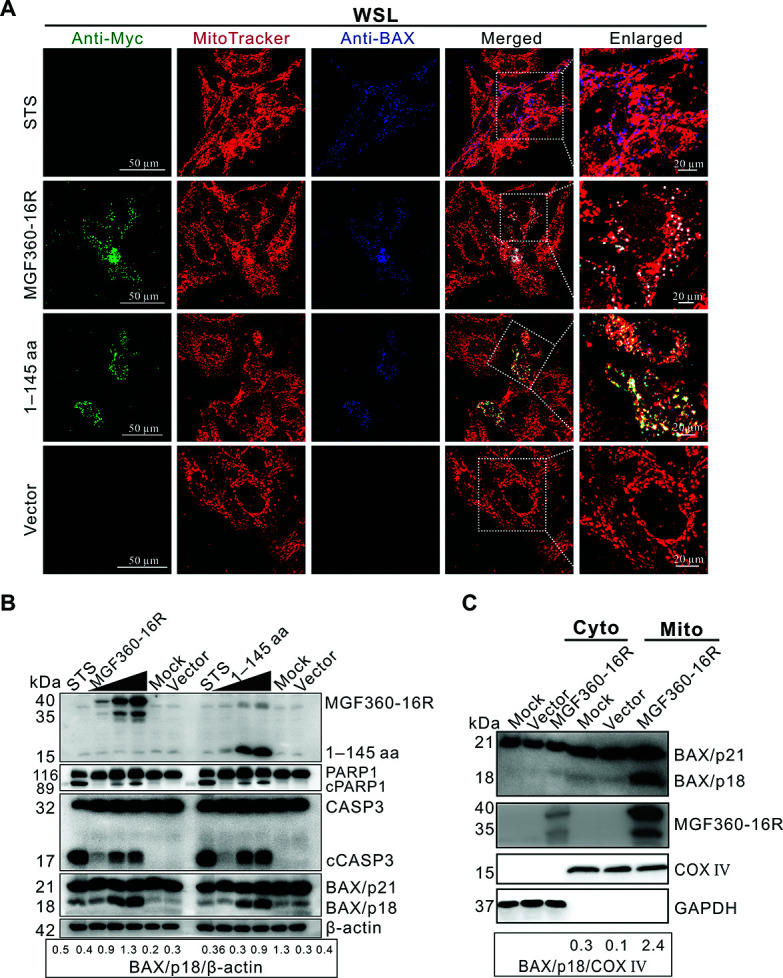
MGF360-16R protein induces mitochondrial-dependent apoptosis by activating BAX. (**A**) WSL was transfected with either an empty vector (pcDNA3.1) or each of its recombinant plasmids expressing Myc-tagged MGF360-16R or truncation 1–145 aa for 36 h. The cells were first stained with MitoTracker Red CMXRos 568 and then analyzed by confocal immunofluorescence using rabbit anti-Myc pAb and mouse anti-BAX mAb as the primary antibodies. The enlarged image of the area within the white-dashed box is provided on the right. Cells treated with 2 µM of STS for 4 h were used as a positive control for apoptosis activation. (**B**) WSL was transfected with either pcDNA3.1 or 1, 2, and 3 µg of recombinant plasmids expressing Myc-tagged MGF360-16R or truncation 1–145 aa, respectively, for 36 h. Then the cells were detected by immunoblotting using antibodies against Myc, PARP1, CASP3, BAX, and β-actin. (**C**) WSL was transfected with either pcDNA3.1 or its recombinant plasmid expressing Myc-tagged MGF360-16R for 36 h. Then the cytosolic (Cyto) and mitochondrial (Mito) fractions of the cells were separated and detected by immunoblotting using antibodies against BAX, MGF360-16R, COX IV, and GAPDH. Representative results from experiments performed in three independent biological replicates are shown.

The activation of pro-apoptotic protein BAX is an important event causing mitochondrial-dependent apoptosis (17, 18). Under homeostatic conditions, BAX is present in cells in its full-length form (BAX/p21), and upon activation, BAX oligomerizes to mitochondria in its active form (BAX/p18), resulting in increased mitochondrial membrane permeability and release of cytochrome c (Cyt c), thereby causing mitochondrial-dependent apoptosis (19). To verify whether MGF360-16R can activate BAX, recombinant plasmids expressing full-length MGF360-16R or its truncation 1–145 aa were transfected into WSL and analyzed by confocal immunofluorescence. Similar to STS-treated WSL, both full-length MGF360-16R and truncation 1–145 aa caused a distinct punctate distribution of BAX in the cytoplasm (Fig. 3A), and a subset of the blue fluorescent puncta of BAX was highly co-localized with the red fluorescent puncta of mitochondria, indicating that MGF360-16R activated BAX on mitochondria via its 1–145 aa. Immunoblotting results further showed that both MGF360-16R and truncation 1–145 aa caused a dose-dependent increase in the activated CASP3 and BAX (Fig. 3B), accompanied by a dose-dependent hydrolysis of nuclear enzyme poly (ADP-ribose) polymerase (PARP-1), which is the substrate of the activated CASP3 (20). In addition, immunoblotting detection of mitochondrial and cytosolic fractions of the transfected WSL showed that BAX activation occurred only in MGF360-16R-transfected WSL, but not in empty vector-transfected cells (Fig. 3C). More specifically, the activated BAX was present only in the mitochondrial fraction and not in the cytosolic fraction (Fig. 3C). These results indicate that MGF360-16R induces apoptosis by activating BAX on mitochondria via its 1–145 aa. Interestingly, the commercial BAX mAb we used could recognize both BAX/p21 and BAX/p18 in the immunoblotting assays, while it could only recognize the activated form of BAX, namely BAX/p18, in the confocal immunofluorescence assays (Fig. 3A). This phenomenon has also been observed in confocal immunofluorescence assays involving the use of this BAX mAb in many papers published by others (21, 22).

**Fig 4:**
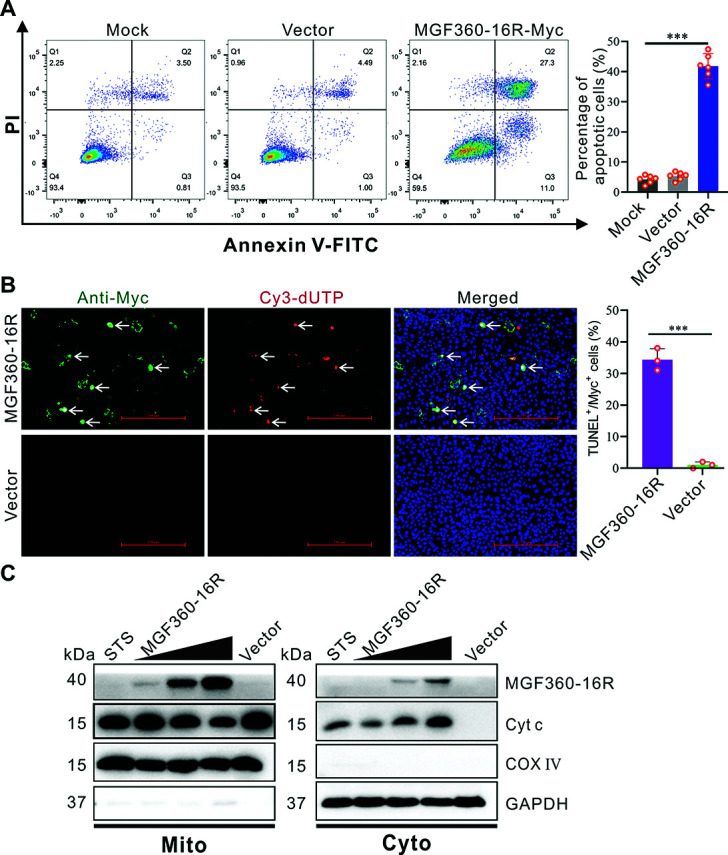
MGF360-16R protein induces apoptosis accompanied by cytochrome c release. (**A**) WSL was mock transfected or transfected with either an empty vector (pcDNA3.1) or a recombinant plasmid expressing Myc-tagged MGF360-16R protein for 36 h. The cells were double-stained with Annexin V-FITC and propidium iodide (PI) and then analyzed by flow cytometry. The percentage of the apoptotic cells is shown as mean ± SD of six independent experiments (Student’s *t*-test; ***P < 0.001). (**B**) WSL was transfected with either pcDNA3.1 or a recombinant plasmid expressing Myc-tagged MGF360-16R protein for 36 h. The cells were fixed and probed with mouse anti-Myc antibody, followed by immunostaining with Alexa Fluor 488-conjugated goat anti-mouse IgG. After counterstaining cell nuclei with DAPI, the cells were stained with TUNEL reagent (Cy3-dUTP) for 2 h at room temperature. The percentage of TUNEL-positive cells in MGF360-16R-positive cells is shown as mean ± SD of three independent experiments (Student’s *t*-test; ***P < 0.001). (**C**) WSL was transfected with either empty vector (pcDNA3.1) or 10, 20, and 30 µg of recombinant plasmid expressing Myc-tagged MGF360-16R for 36 h. The cytosolic (Cyto) and mitochondrial (Mito) fractions of the cells were separated and detected by immunoblotting using antibodies against MGF360-16R, cytochrome c (Cyt c), COX IV, and GAPDH.

Furthermore, MGF360-16R-transfected WSL was also analyzed by flow cytometry combined with Annexin-V-FITC/propidium iodide (PI) double staining. Approximately 41% of MGF360-16R-transfected WSL underwent apoptosis, significantly higher than those in mock cells and empty vector-transfected cells (Fig. 4A). Further *in situ* TUNEL staining of the transfected WSL showed that about 35% of cells expressing MGF360-16R were simultaneously positive for Cy3-dUTP staining (Fig. 4B), confirming that MGF360-16R can induce apoptosis. Since Cyt c release is an important indicator of mitochondrial-dependent apoptosis (23), the mitochondrial and cytosolic fractions of MGF360-16R-transfected WSL were detected by immunoblotting. With the increase in MGF360-16R transfection dose, the amount of Cyt c in mitochondrial fractions gradually decreased, while that in the corresponding cytosolic fractions gradually increased (Fig. 4C), indicating that MGF360-16R caused the release of Cyt c. Taken together, MGF360-16R functions to induce mitochondrial-dependent apoptosis by activating BAX on mitochondria.

### BAX is crucial for MGF360-16R-induced apoptosis

**Fig 5:**
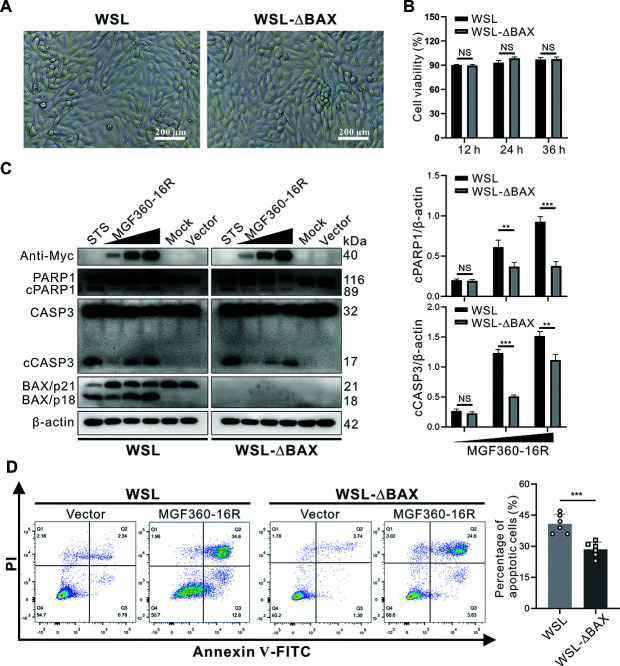
BAX is essential to MGF360-16R-induced apoptosis. (**A**) The appearance of wild-type WSL and WSL-ΔBAX cells. (**B**) Cell viability analysis of WSL and WSL-ΔBAX cells by CCK-8 assay. The cell’s vitality is shown as mean ± SD of three independent experiments (Student’s t-test; NS *P* > 0.05). (**C**) WSL and WSL-ΔBAX cells were transfected with either pcDNA3.1 or 1, 2, and 3 µg of recombinant plasmid expressing Myc-tagged MGF360-16R, respectively, for 36 h. Then the cells were detected by immunoblotting using antibodies against Myc, PARP1, CASP3, BAX, and β-actin. Representative results from experiments performed in three independent biological replicates are shown. (**D**) WSL and WSL-ΔBAX cells were mock transfected or transfected with either an empty vector (pcDNA3.1) or a recombinant plasmid expressing Myc-tagged MGF360-16R protein for 36 h. The cells were double-stained with Annexin V-FITC and propidium iodide (PI) and then analyzed by flow cytometry. The percentage of the apoptotic cells is shown as mean ± SD of six independent experiments (Student’s t-test; ***P < 0.001).

To investigate whether BAX is essential to MGF360-16R-induced apoptosis, we constructed a monoclonal BAX-knockout WSL cell line (designated WSL-ΔBAX) and analyzed the effect of BAX depletion on the ability of MGF360-16R to induce apoptosis by immunoblotting and flow cytometry. Before the formal experiments, we first measured cell viability to rule out the possible impact of BAX depletion on the viability of WSL cells, which, in turn, affects their ability to undergo apoptosis. As shown in Fig. 5A and B, BAX depletion had no significant effect on the appearance and viability of WSL cells. The results of immunoblotting showed that the levels of CASP3 activation and PARP1 cleavage caused by MGF360-16R transfection in WSL-ΔBAX cells were significantly lower than those in wild-type WSL cells (Fig. 5C), which was further confirmed by flow cytometry analysis using Annexin-V/PI double staining (Fig. 5D). Overall, these results suggest that BAX is required for MGF360-16R-mediated apoptosis.

### MGF360-16R protein is localized to the viral factory early in infection and partially translocated to mitochondria later in infection

**Fig 6:**
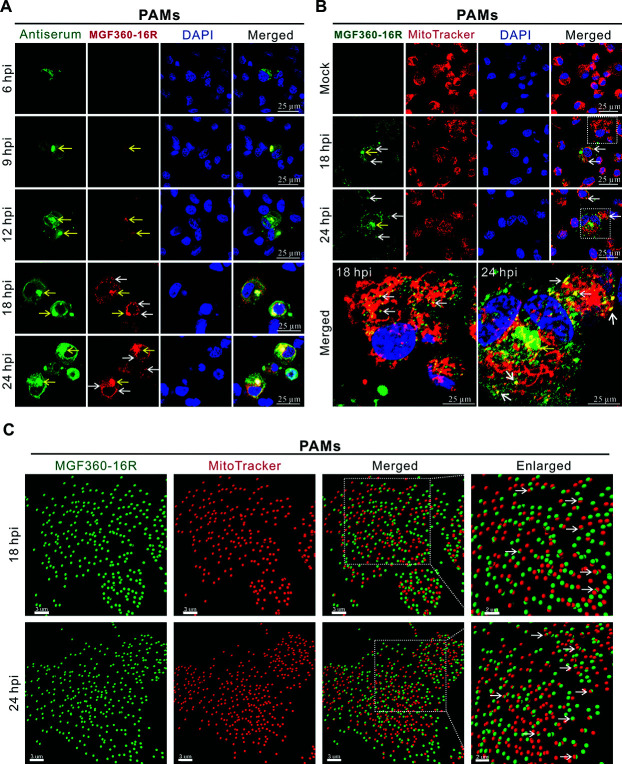
MGF360-16R protein is localized to the viral factory early in infection and partially translocated to mitochondria later in infection. (**A**) PAMs were mock infected or infected with ASFV-HN09 at an MOI of 2. At the indicated times, PAMs were fixed and incubated with mouse anti-MGF360-16R pAb and swine ASFV antiserum, followed by immunostaining with Alexa Fluor 568-conjugated goat anti-mouse IgG and FITC-conjugated goat anti-swine IgG, respectively. After counterstaining cell nuclei with DAPI, the cells were observed by a confocal microscope. The representative localization of MGF360-16R protein inside and outside the viral factory is indicated by yellow and white arrows, respectively. (**B**) PAMs were infected as described in (**A**). At 18 and 24 hpi, PAMs were stained with MitoTracker Red CMXRos 647 for 30 min at 37°C before fixation. Then the cells were incubated with mouse anti-MGF360-16R pAb, followed by immunostaining with Alexa Fluor 568-conjugated goat anti-mouse IgG. After counterstaining cell nuclei with DAPI, the cells were observed by a confocal microscope. The representative localization of MGF360-16R protein inside and outside the viral factory is indicated by yellow and white arrows, respectively. The enlarged image of the area within the white-dashed box is provided below. (**C**) Three-dimensional reconstruction of co-localization between MGF360-16R protein and mitochondria. The areas within the white-dashed boxes shown in (**B**) were reconstructed by Imaris software.

Our above results indicate that MGF360-16R is localized to mitochondria under transfection conditions, but whether this is also the case under infection conditions remains unknown. We then analyzed the spatiotemporal distribution of MGF360-16R in ASFV-infected cells by confocal immunofluorescence. The expression of MGF360-16R was detectable in ASFV-infected PAMs from 9 h post-infection (hpi) onward, and its expression level gradually increased as ASFV infection progressed (Fig. 6A). The red immunostaining signals of MGF360-16R were mainly distributed in large puncta or clumps in the cytoplasm of ASFV-infected PAMs, which were highly co-localized not only with the green punctate immunostaining signals of ASFV antiserum but also with the blue staining signals of DNA in large puncta distributed around the nucleus. This suggests that under ASFV infection conditions, at least in the early stages of infection, MGF360-16R is mainly localized to the viral factory (indicated by yellow arrows), which is a special structure containing ASFV structural proteins and viral DNA distributed around the nucleus (24, 25). The temporal dynamic analysis further revealed that this phenomenon could last up to 12 hpi. However, starting from 18 hpi, except for a small amount of MGF360-16R still distributed in clumps in the viral factory (indicated by yellow arrows), the majority of MGF360-16R was distributed in distinct puncta in the cytoplasm (indicated by white arrows) (Fig. 6A), which is consistent with the distribution of MGF360-16R under transfection conditions (Fig. 3A). This phenomenon implies that the localization of MGF360-16R may be altered during ASFV infection. To verify this, the ASFV-infected PAMs at 18 hpi and 24 hpi were additionally stained for mitochondria. The green staining signals of MGF360-16R were mainly characterized by a cytoplasmic punctate distribution (indicated by white arrows), although a small amount of clumpy distribution could still be observed (indicated by yellow arrows). More importantly, most of the green fluorescent puncta of MGF360-16R could co-localize with the red staining signals of mitochondria, which appeared as yellow puncta after merging (Fig. 6B). Further three-dimensional (3D) reconstruction revealed that a subset of MGF360-16R was indeed co-localized with the mitochondria (Fig. 6C). Similar results were also obtained in ASFV-infected WSL (Fig. S3). Moreover, the mitochondrial and cytosolic fractions separated from ASFV-infected PAMs were detected by immunoblotting. The results showed that MGF360-16R was detectable in both the cytoplasmic and mitochondrial fractions of ASFV-infected PAMs at 24 hpi (Fig. S4). Notably, the translocation of MGF360-16R to mitochondria was only prominent in the floating cells but not the adherent cells. The reason for this phenomenon is believed to be that ASFV infection caused a large number of apoptotic cells to transition from an adherent state to a floating state, especially in the late stage of ASFV infection. More activated BAX/p18 was detected from the floating cells (Fig. S4), confirming that ASFV infection could cause a large number of apoptotic cells to float. Taken together, our results demonstrate that MGF360-16R protein is mainly localized to the viral factory in the early stage and then shifts a portion to mitochondria in the late stage.

### MGF360-16R protein activates BAX by competitively interacting with the HSP60 protein

**Fig 7:**
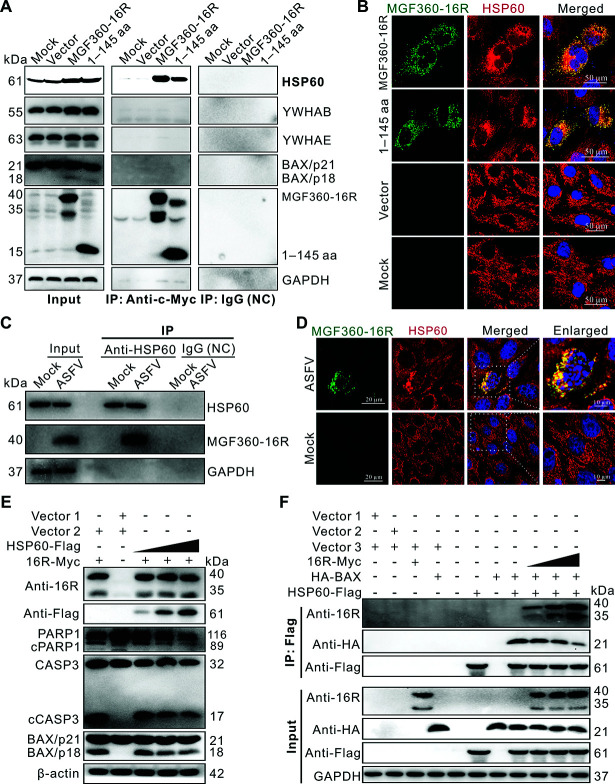
MGF360-16R protein activates BAX by competing with BAX to bind to HSP60 protein. (**A**) WSL was transfected with either an empty vector (pcDNA3.1) or its recombinant plasmids expressing Myc-tagged MGF360-16R or truncation 1–145 aa for 36 h. The cell lysates were analyzed by Co-IP assays using anti-Myc magnetic beads and with the antibodies against HSP60, YWHAB, YWHAE, BAX, MGF360-16R, and GAPDH. A mouse isotype IgG1 was used as a control. (**B**) WSL was transfected as described in (**A**) for 24 h. The cells were analyzed by confocal immunofluorescence using antibodies against MGF360-16R and HSP60. (**C**) Co-IP analysis of the interaction between endogenous HSP60 and MGF360-16R in ASFV-infected primary PAMs at 48 hpi (MOI = 2). A rabbit isotype IgG was used as a control. (**D**) Confocal immunofluorescence analysis of the interaction between endogenous HSP60 and MGF360-16R in ASFV-infected WSL at 24 hpi (MOI = 2). The enlarged image of the area within the white-dashed box is provided on the right. (**E**) WSL was co-transfected with recombinant plasmids expressing Myc-tagged MGF360-16R and Flag-tagged HSP60 or their respective empty vectors (vector 1: pcDNA3.1-Myc; vector 2: pcDNA3.1-Flag) for 36 h. The cell lysates were analyzed by immunoblotting using antibodies against MGF360-16R, Flag, PARP1, CASP3, BAX, and β-actin. (**F**) WSL was co-transfected with recombinant plasmids expressing Myc-tagged MGF360-16R, Flag-tagged HSP60, and HA-tagged BAX or their respective empty vectors (vector 1: pcDNA3.1-Myc; vector 2: pcDNA3.1-Flag; vector 3: pCMV-HA) for 36 h. The cell lysates were analyzed by Co-IP assays using anti-Flag magnetic beads and with the antibodies against MGF360-16R, HA, Flag, and GAPDH. Representative results (**A, C, E, F**) from experiments performed in three independent biological replicates are shown.

The key host proteins interacting with MGF360-16R were screened by co-immunoprecipitation (Co-IP) assays combined with mass spectrometry using cell lysates extracted from MGF360-16R-transfected WSL. In total, four cellular proteins potentially interacting with MGF360-16R were obtained, including heat shock protein 60 (HSP60), BAX, YWHAB, and YWHAE. Of them, only the HSP60 protein co-immunoprecipitated with MGF360-16R and its truncation 1–145 aa, while BAX, YWHAB, and YWHAE did not (Fig. 7A), indicating that MGF360-16R interacts with HSP60 through its N-terminal 1–145 aa domain. Confocal immunofluorescence further showed that both full-length MGF360-16R and its truncation 1–145 aa were highly co-localized with HSP60 in transfected WSL, confirming that MGF360-16R interacts with HSP60 via its 1–145 aa (Fig. 7B). Furthermore, Co-IP assays were conducted to investigate whether MGF360-16R and HSP60 proteins also interact under ASFV infection. The results showed that MGF360-16R was still able to interact with HSP60 in ASFV-infected PAMs (Fig. 7C). In addition, confocal immunofluorescence further showed that MGF360-16R and HSP60 were highly co-localized in the cytoplasm of ASFV-infected WSL (Fig. 7D), confirming that the two proteins can interact under infection conditions.

HSP60 protein mainly exists in mitochondria by forming the HSP60-BAX complex with BAX, thereby limiting BAX’s pro-apoptotic effect. Reduction of HSP60 can cause BAX activation, which, in turn, triggers apoptosis (26). Based on this, we speculated that MGF360-16R may compete with BAX to bind HSP60 in the HSP60-BAX complex, causing the release and activation of BAX upon dissociation from the complex. To verify this, MGF360-16R and HSP60 proteins were co-expressed in WSL, followed by immunoblotting analysis. While keeping the same MGF360-16R protein input, the level of mitochondrial-dependent apoptosis in WSL gradually decreased with the increase in HSP60 protein input, as evidenced by a gradual decrease in CASP3 and BAX activations and PARP1 hydrolysis (Fig. 7E). This indicates that the increase in HSP60 can inhibit MGF360-16R-mediated BAX activation and mitochondrial-dependent apoptosis. Next, to clarify how MGF360-16R activates BAX by interacting with HSP60, the competitive relationship of MGF360-16R, HSP60, and BAX in co-transfected WSL was analyzed by Co-IP assays. While maintaining the same amount of HSP60 and BAX protein input, as the input of MGF360-16R protein gradually increased, the amount of BAX protein in the immune complex pulled down by anti-Flag magnetic beads gradually decreased (Fig. 7F). This indicates that MGF360-16R competes with BAX to interact with HSP60. In addition, the competitive binding relationship between MGF360-16R, HSP60, and BAX was further analyzed by confocal immunofluorescence. As shown in Fig. S5, in the double-transfected WSL, whether co-expressing MGF360-16R and HSP60, or co-expressing HSP60 and BAX, any two co-expressed proteins exhibited a distinct punctate cytoplasmic distribution, and their fluorescent puncta were well co-localized. After merging, the red MGF360-16R fluorescent puncta and the green HSP60 fluorescent puncta became yellow puncta (the upper panels), confirming the interaction between MGF360-16R and HSP60; the green HSP60 fluorescent puncta, and the blue BAX fluorescent puncta became light blue puncta (the middle panels), implying that HSP60 and BAX exist in the form of the complex. Notably, in the triple-transfected WSL, when MGF360-16R, HSP60, and BAX were co-expressed, MGF360-16R and HSP60 remained highly co-localized, and both showed co-localization with the majority of BAX (the lower panels), displaying white fluorescent puncta in the merged images. However, a subset of BAX no longer co-localized with MGF360-16R and HSP60 (the lower panels), displaying scattered blue fluorescent puncta in the merged images. These findings suggest that MGF360-16R competes with BAX for binding to HSP60, causing the release of BAX from the HSP60-BAX complex. Altogether, these results indicate that MGF360-16R activates BAX by competitively interacting with HSP60.

### The 433–573 aa of HSP60 is the competitive binding domain with MGF360-16R and BAX

**Fig 8:**
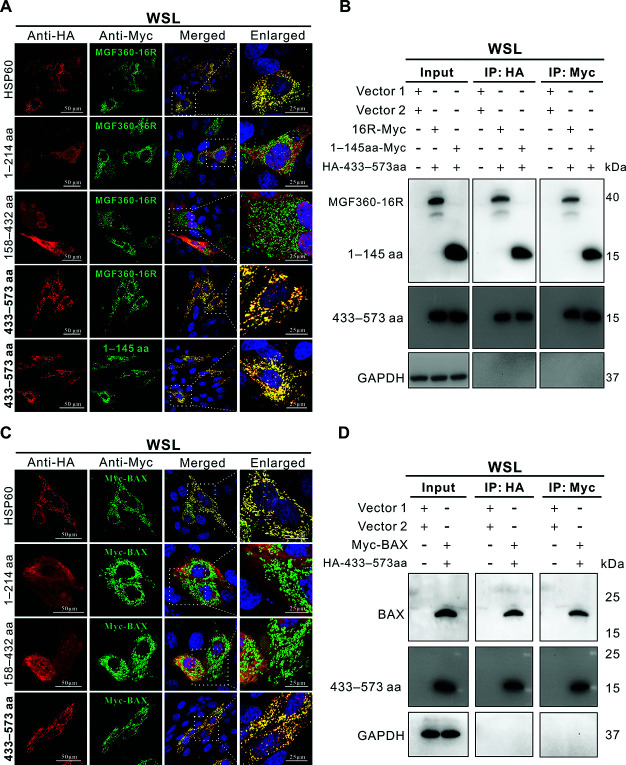
The 433–573 amino acids of HSP60 protein are the competitive binding domain with MGF360-16R and BAX. (**A**) WSL was co-transfected with recombinant plasmids expressing Myc-tagged MGF360-16R or its truncation 1–145 aa and recombinant plasmids expressing HA-tagged HSP60 or each of its truncations (1–214 aa, 158–432 aa, and 433–573 aa) or their respective empty vectors for 24 h. The cells were incubated with HA and Myc antibodies, followed by immunostaining with Alexa Fluor 568-conjugated goat anti-mouse IgG and 488-conjugated goat anti-rabbit IgG, respectively. After counterstaining cell nuclei with DAPI, the cells were observed by a confocal microscope. The enlarged image of the area within the white-dashed box is provided on the right. (**B**) WSL was co-transfected as described in (**A**) and then analyzed by reciprocal Co-IP assays (Vector 1: pcDNA3.1-Myc, vector 2: pCMV-HA). (**C**) WSL was co-transfected with recombinant plasmids expressing HA-tagged HSP60 or each of its truncations (1–214 aa, 158–432 aa, and 433–573 aa) and recombinant plasmids expressing Myc-tagged BAX or their respective empty vectors for 24 h. The cells were incubated with HA and Myc antibodies, followed by immunostaining with Alexa Fluor 568-conjugated goat anti-mouse IgG and 488-conjugated goat anti-rabbit IgG, respectively. After counterstaining cell nuclei with DAPI, the cells were observed by a confocal microscope. The enlarged image of the area within the white-dashed box is provided on the right. (**D**) WSL was co-transfected as described in (**C**), and then analyzed by reciprocal Co-IP assays (Vector 1: pCMV-Myc, vector 2: pCMV-HA).

To identify the key functional domain of HSP60 responsible for interacting with MGF360-16R, either full-length HSP60 or each of its three overlapping truncations (1–214 aa, 158–432 aa, and 433–573 aa) was transfected into WSL with full-length MGF360-16R or truncation 1–145 aa, followed by confocal immunofluorescence analysis. Both the red immunostaining fluorescence of HSP60 and the green immunostaining fluorescence of MGF360-16R showed a punctate cytoplasmic distribution and were highly co-localized (Fig. 8A). Of the three HSP60 truncations, only truncation 433–573 aa still displayed discrete punctate red fluorescent signals, similar to the full-length HSP60. More importantly, the red fluorescent puncta of truncation 433–573 aa were highly co-localized not only with the green fluorescent puncta of full-length MGF360-16R but also with those of truncation 1–145 aa (Fig. 8A). This indicates that the 433–573 aa of HSP60 and 1–145 aa of MGF360-16R are the domains mediating their interaction. Reciprocal Co-IP assays showed that the HSP60 truncation 433–573 aa pulled down both MGF360-16R and its truncation 1–145 aa and vice versa (Fig. 8B). These results confirm that the HSP60 433–573 aa and the MGF360-16R 1–145 aa are the key interaction domains between the two proteins. In addition, whether HSP60 uses the same domain to bind to BAX was also analyzed. The confocal immunofluorescence results showed that only the truncation 433–573 aa, like the full-length HSP60, exhibited red fluorescent puncta evenly distributed in the cytoplasm, which was highly co-localized with the green fluorescent puncta of BAX (Fig. 8C). This reveals that HSP60 protein interacts with BAX through its 433–573 aa domain, which was confirmed by the reciprocal Co-IP results (Fig. 8D).

### Knockout of MGF360-16R reduces the ability of ASFV to induce apoptosis and replication

**Fig 9:**
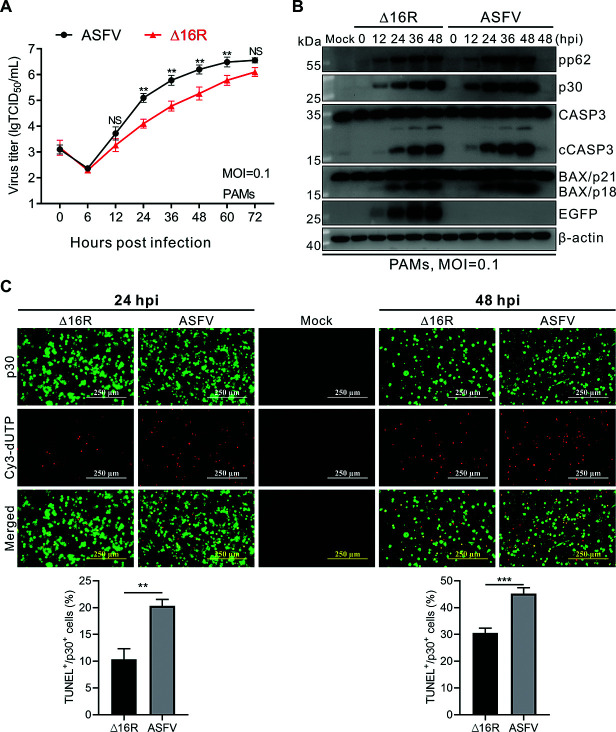
Knockout of MGF360-16R reduces the ability of ASFV to induce apoptosis and replication. (**A**) Growth kinetics of ASFV HN09 and ∆16R in PAMs at an MOI of 0.1. (**B**) Primary PAMs were mock infected or infected with ASFV HN09 or ∆16R at an MOI of 0.1. At the indicated times, PAMs were detected by immunoblotting using antibodies against pp62, p30, GFP, CASP3, BAX, and β-actin. Representative results from experiments performed in three independent biological replicates are shown. (**C**) Primary PAMs were mock infected or infected with ASFV HN09 or ∆16R at an MOI of 1. At 24 and 48 hpi, PAMs were fixed and probed with mouse anti-p30 mAb, followed by immunostaining with Alexa Fluor 488-conjugated goat anti-mouse IgG. After counterstaining cell nuclei with DAPI, the cells were stained with TUNEL reagent (Cy3-dUTP) for 2 h at room temperature. The percentage of TUNEL-positive cells in p30-positive cells is shown as mean ± SD of three independent experiments (Student’s t-test; ***P < 0.001).

To further validate the role of MGF360-16R in apoptosis induction and ASFV replication, we constructed an MGF360-16R-knockout ASFV mutant (designated ∆16R) using the construction strategy shown in Fig. S6A. PCR identification of the parental ASFV and Δ16R showed that when using a specific primer pair F1/R1 for the MGF360-16R gene, a fragment of the expected size could be amplified from the genome of the parental ASFV but not from Δ16R (Fig. S6B). By contrast, when a specific primer pair F2/R2 for the EGFP gene was used, a fragment of the expected size could be amplified from the genome of Δ16R but not from the genome of the parental ASFV (Fig. S6B). This reveals that Δ16R was successfully purified. Next, to ensure the successful construction of Δ16R, the genomic DNA from the fifth-generation purified Δ16R was extracted for next-generation sequencing (NGS) analysis. The results showed that the MGF360-16R gene, originally located between the DP238L and MGF505-11L genes, was successfully knocked out from the Δ16R genome (Fig. S6C), as demonstrated by the construction strategy shown in Fig. S6A. Specifically, the MGF360-16R gene was accurately deleted from the genome of ∆16R and replaced by a cassette containing an EGFP reporter gene under the control of the ASFV p72 promoter (Fig. S6D). It should be pointed out that in addition to the deletion of the MGF360-16R gene, the Δ16R genome also unexpectedly produced four base mutations. Among them, two mutations (10386 [T→C] and 139582 [G→A]) are located in the non-coding region (NCR), while the other two mutations (61782 [A→G] and 166823 [T→C]), although located in the F1055L and E199L genes, respectively, are synonymous. Accordingly, we believe that these four additional mutations will not affect the function of Δ16R in inducing apoptosis. Determination of viral growth kinetics in PAMs showed that although their proliferation trends were similar, the titer of parental ASFV was significantly higher than that of ∆16R from 24 hpi onward, with titer differences of 10, 10, 9.3, and 7.1 times at 24, 36, 48, and 60 hpi, respectively (Fig. 9A), indicating that deletion of MGF360-16R weakens the replication of ASFV. Subsequently, both parental ASFV and ∆16R were used to synchronously infect PAMs for immunoblotting analysis. The results showed that although both parental ASFV and ∆16R exhibited a similar trend in inducing the activation of CASP3 and BAX in PAMs, as manifested by a progressive increase in CASP3 and BAX activation from 12 hpi onward, the activation ability of ∆16R was lower than that of parental ASFV at each time point (Fig. 9B). Furthermore, *in situ* TUNEL staining of the infected PAMs showed that the ratio of Cy3-dUTP-positive cells to p30-positive cells at 24 hpi and 48 hpi was 11.9% and 30.5% in the ∆16R-infected group, respectively, which is significantly lower than that in the parental ASFV-infected group (20.3% and 45.2%) (Fig. 9C). This indicates that the ability of ∆16R to induce apoptosis is weaker than that of the parental strain. The above-mentioned results indicated that the deletion of MGF360-16R weakened the ability of ASFV to induce apoptosis, but did not completely abolish the ability. This suggests that MGF360-16R does have the function of inducing mitochondrial-dependent apoptosis, but there should be other apoptosis-inducing proteins in ASFV. Notably, the expression level of pp62 and p30 proteins in ∆16R-infected PAMs was lower than that of parental ASFV at each time point, indicating that deletion of MGF360-16R did not affect the survival of ASFV, but weakened the replication of ASFV (Fig. 9B). These results suggest that although MGF360-16R is not required for ASFV replication, its absence adversely affects ASFV replication.

## DISCUSSION

**Fig 10:**
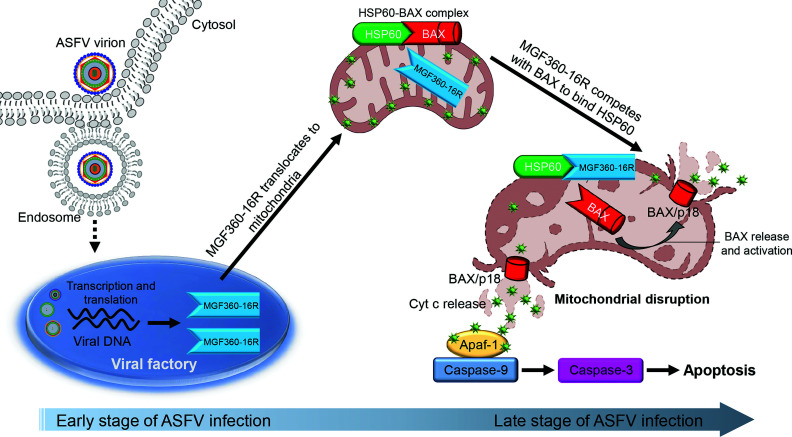
A proposed a model for the mitochondrial-dependent apoptosis induced by the ASFV MGF360-16R protein. MGF360-16R protein activates BAX in the HSP60-BAX complex by competing with BAX to bind to HSP60, thereby activating mitochondrial-dependent apoptosis.

Although cell apoptosis related to ASFV infection has been described for decades, the underlying mechanisms have not been fully understood so far. Prior to this study, only a few ASFV proteins had been sporadically reported to induce apoptosis (12, 15, 16). Given the large genome and numerous coding proteins, there should be other proteins in ASFV that can induce apoptosis. In this study, we used immunoblotting detection of caspase-3 activation as a tool for genome-scale screening of ASFV-encoded apoptosis inducers. Ultimately, we successfully screened a total of 27 ASFV proteins with the potential to induce apoptosis (Fig. 1A; Table S1). Among them, the MGF360-16R protein was selected for functional research due to its strong activation of caspase-3 and most pronounced co-localization with mitochondria. Our final results revealed that MGF360-16R functions as an inducer of mitochondrial-dependent apoptosis through a mechanism that competitively binds to the HSP60 protein with BAX. A model for MGF360-16R-induced mitochondrial-dependent apoptosis is proposed in Fig. 10.

Caspase-3 is one of the key effectors responsible for the execution of apoptosis, which can be activated by both intrinsic and extrinsic pathways (27–29). In addition, mitochondria have been demonstrated to act as amplifiers of caspase activities. Caspases upstream of the apoptotic pathway are known to affect mitochondrial events in both intrinsic and extrinsic apoptosis pathways, either directly or indirectly, through interactions with members of the Bcl-2 family (30, 31). Therefore, the activation of caspase-3 was used as an indicator for screening of apoptosis-inducing ASFV proteins. It is worth noting that among the three ASFV proteins (p54, E199L, and CD2v) previously reported to induce apoptosis (12, 15, 16), p54 was also screened out by our method. The reason why E199L and CD2v proteins were not screened by our method might be caused by the different screening systems used in these studies. Our system used immunoblotting to detect caspase-3 activation in transfected WSL, while their screening system used a CellTiter-Glo luminescent assay to analyze the cell activity of transfected HEK293T cells (15). The main advantage of our system is that ASFV proteins can be screened whether they induce apoptosis of intrinsic or extrinsic pathways. However, our system also has some disadvantages, such as being relatively laborious and not applicable to screen ASFV proteins that induce apoptosis of non-permissive bystander cells.

Previous studies have shown that ASFV can induce apoptosis of not only target cells of the mononuclear phagocyte system but also bystander non-susceptible cells, especially lymphocytes (7, 9). From the current research progress, the biological significance of apoptosis induced by ASFV is mainly reflected in two aspects. On the one hand, the induction of apoptosis during infection is an important mechanism by which ASFV causes immunosuppression. It has been reported that both ASFV-susceptible monocytes and macrophages and non-susceptible immunocompetent cells can undergo apoptosis due to ASFV infection (32–34). Because these cells are important immunocytes that perform innate and acquired immune functions in pigs, their apoptosis will inevitably reduce the effectiveness of the host immune response to ASFV infection and lead to damage to multiple organs including immune organs, thus contributing to the pathogenesis of ASFV (7, 34). In this study, we demonstrated for the first time that the ASFV MGF360-16R protein also functions as an apoptosis inducer, which can induce apoptosis independently. This further enriches our knowledge about the causative properties of ASFV. On the other hand, the induction of apoptosis contributes to the timely release of progeny viruses from the infected target cells, favoring ASFV dissemination but avoiding the induction of inflammatory signals that can activate an immune response to clear the infection (34). One of our recent studies demonstrated that ASFV can produce apoptotic bodies containing single-enveloped but infectious ASFV particles at the late stage of infection, which are used as a delivering vehicle for cell-to-cell transmission, thereby enabling viral invasion and immune escape (14). Overall, apoptosis induced by ASFV can be deemed as an important component of ASFV pathogenesis.

Given the important roles that apoptosis plays in the course of ASFV infection, investigating the underlying mechanisms of ASFV-induced apoptosis will help to understand ASFV pathogenesis and develop effective vaccine strategies. The ASFV p54 protein was demonstrated to induce mitochondrial-dependent apoptosis by directly binding to the light chain of dynein through a p54-binding motif similar to that of BIM-3, a member of the pro-apoptotic BCL-2 family, suggesting that p54 might induce apoptosis by replacing Bim-3 from microtubules (12, 35). Similarly, the ASFV E199L protein was proved to induce mitochondrial-dependent apoptosis by activating BAK and BAX by competing with BAK to bind to BCL-xL (15). In the present study, we demonstrated that the MGF360-16R protein is capable of inducing mitochondrial-dependent apoptosis by competing with BAX to bind to HSP60. These findings suggest that ASFV should synergistically regulate apoptosis through multiple proteins, including those that have not yet been identified. Notably, the results of two recent studies on the role of ASFV CD2v protein in regulating apoptosis are opposite. One study showed that CD2v can induce apoptosis in swine peripheral blood mononuclear cells and macrophage cultures (16), while another study found that CD2v inhibits apoptosis by interacting with host CSF2RA to activate the JAK2-STAT3 pathway (36). Although the reason for the opposite results remains unknown, we speculate that it may be related to the different strains used in the experiments. The former used the genotype I Congo K-49 strain, while the latter used the genotype II GZ201801 strain. In addition, endoplasmic reticulum stress-induced apoptosis was also confirmed to be involved in the process of apoptosis caused by ASFV infection, playing a role conducive to virus transmission (34). The mechanisms of ASFV-induced apoptosis mentioned above were mainly focused on ASFV-infected permissive cells; however, ASFV can also induce apoptosis of the non-susceptible bystander cells. By contrast, less is known about the mechanisms by which ASFV induces apoptosis of bystander cells. It has been reported that cytokines or chemicals secreted by ASFV-infected target cells such as macrophages may be a mechanism to mediate apoptosis of non-susceptible bystander cells (34, 37).

So far, little is known about the function of the MGF360-16R protein. Only one published paper reported that the MGF360-16R protein of the ASFV Georgia strain could interact with the host proteins SERTAD3 and SDCBP, both of which are involved in nuclear transcription, and SDCBP also participates in virus traffic inside the host cells (38). Therefore, it was inferred that MGF360-16R might be involved in nuclear transcription and viral transport within host cells, despite further validation being needed. Notably, although the study further claimed that the MGF360-16R-deleted Georgian strain (ASFV-G-ΔMGF360-16R) they constructed had the same replication ability in primary swine macrophage cells and virulence in domestic pigs as the parental strain ASFV-G, the viremia titers of pigs infected with ASFV-G-ΔMGF360-16R were lower than those infected with the parental strain within 1–7 days post-challenge, with a significant difference appearing on the fourth day post-challenge (38). Overall, their results are basically consistent with those of our present study, although the ASFV strains used in the two studies were different, and there might be potential strain differences. In addition, it should be pointed out that it is indeed difficult for us to conclude whether the reduced apoptosis-inducing ability of the MGF360-16R-knockout strain is caused by the decreased replication capacity of the virus or by the weakened apoptosis-inducing ability of the MGF360-16R-knockout strain. Nevertheless, our current study demonstrated for the first time that MGF360-16R functions as a mitochondrial-dependent apoptosis inducer by competing with BAX to bind to the HSP60 protein. Our results not only reveal a new function of MGF360-16R protein but also deepen our understanding of the pathogenesis of ASFV.

## MATERIALS AND METHODS

### Bio-safety and ethics approval

All experiments involving ASFV operation were conducted in the Biosafety Level 3 (P3) Laboratory of China Agricultural University in strict accordance with the relevant biosafety regulations and operating procedures of the P3 laboratory (Approval No. 2022-ASFV-002). Preparation of primary PAMs from SPF pigs was performed as described previously (39), under the approval of the Laboratory Animal Welfare and Animal Experimental Ethical Committee of China Agricultural University (Approval No. AW11402202-2-1).

### Viruses and cells

The ASFV CADC_HN09 strain (GenBank accession no. MZ614662.1) was provided by the China Animal Disease Control Center (Beijing, China). The WSL cell line that supports efficient replication of ASFV was obtained through four rounds of sub-cloning screening in our laboratory. Both PAMs and WSL were cultured with RPMI 1640 supplemented with 10% fetal bovine serum, 100 U/mL penicillin, and 100 µg/mL streptomycin at 37°C with 5% CO_2_. A monoclonal BAX-knockout WSL cell line was constructed by Genomeditech (Shanghai) Co., Ltd. Briefly, WSL was first infected with a packaged Cas9-Blast (GM-LV3919) lentivirus to generate a stable WSL cell line expressing Cas9, which was then infected with another lentivirus expressing Sus scrofa sgRNA (GM-77116LV) to produce BAX-knockout WSL. Using blasticidin and puromycin screening, a monoclonal BAX-knockout WSL cell line was finally obtained and designated WSL-∆BAX.

### Antibodies and reagents

MGF360-16R polyclonal antibody (pAb), pp62, and p30 mAbs were prepared in mice, and swine ASFV antiserum was preserved in our laboratory (14). Mouse anti-HA (M180) and Flag (M185) mAbs were purchased from MBL Beijing Biotech Co., Ltd. Rabbit anti-GAPDH (10494–1-AP), PARP1 (13371–1-AP), YWHAE (11648–2-AP), HSP60 (15282–1-AP), GFP (50430–2-AP), Tom20 (11802–1-AP) and Myc (16286–1-AP) pAbs, and mouse anti-Myc (60003–2-Ig), YWHAB (66061–1-Ig), BAX (60267–1-Ig), β-actin (66009–1-Ig) mAbs were purchased from Proteintech Group, Inc. Rabbit anti-caspase-3 (14220), cleaved caspase-3 (9664), Cyt c (11940), COX IV (4850), and MitoTracker Red CMXRos 568 (9082) were purchased from Cell Signaling Technology (Boston, MA, USA). Horseradish peroxidase-conjugated goat anti-rabbit (ZB-2301) and anti-mouse (ZB-2305) IgGs were purchased from ZSGB-BIO. MitoTracker Red CMXRos 647 (M22426), Lipofectamine LTX reagent (A12621), Lipofectamine RNAiMAX reagent (13778150), DAPI (62248), Alexa Fluor 568-conjugated goat anti-mouse F(ab′)2 fragment (A11019), Alexa Fluor 488-conjugated goat anti-mouse F(ab′)2 fragment (A11017), Alexa Fluor 488-conjugated goat anti-rabbit F(ab′)2 fragment (A11070), Alexa Fluor 647-conjugated goat anti-mouse F(ab′)2 fragment (A21237), and Alexa Fluor 568-conjugated goat anti-rabbit F(ab′) fragment (A11011) were obtained from Thermo Fisher Scientific. Fluorescein (FITC)-conjugated goat anti-swine IgG (H + L) (114-095-003) was purchased from Jackson ImunnoResearch. Anti-Myc magnetic beads (HY-K0206), Anti-HA magnetic beads (HY-K0201), anti-Flag magnetic beads (HY-K0207), and STS (HY-15141) were purchased from MedChem Express. TUNEL apoptosis detection kit (C1090) was purchased from Beyotime Biotechnology. Annexin V-FITC/PI apoptosis detection kit (A211-01) was purchased from Vazyme Biotech Co., Ltd. Mitochondrial isolation and purification kit (MP-007) was purchased from Invent Biotechnologies, Inc. Protein A/G PLUS-Agarose (sc-2003) was purchased from Santa Cruz Biotechnology. X-tremeGENE HP DNA transfection agent was purchased from Roche, XTGHP-RO. Cell counting kit-8 (CCK-8; CK04) was purchased from Dojindo Laboratories.

### Virus infection and titration

PAMs and WSL were mock infected or infected with ASFV-HN09 at an MOI of 0.1 or 2. At the specified times, the cells together with supernatants were freeze-thawed thrice. After removal of cell debris by centrifugation, the virus titer was determined on PAMs using a microtitration infectivity assay, and the results were interpreted by an indirect immunofluorescence assay based on p30 antibody staining (40). The titer was reported as 50% tissue culture infective dose per milliliter (TCID_50_/mL) according to the Reed–Muench method.

### Plasmid construction

The 178 ASFV protein-coding genes listed in Table S1 were synthesized by Beijing Tsingke Biotech Co., Ltd. based on codon optimization with reference to the ASFV HN09 strain and separately cloned into the pcDNA3.1 vector carrying a C-terminal Myc tag. After sequencing to ensure accuracy, they were used for the subsequent experiments. The truncated forms of MGF360-16R, including 1–145 aa, 40–215 aa, and 146–352 aa, were also cloned into pcDNA3.1. The coding gene of the HSP60 protein (GenBank no. NM_001254716.1) was cloned into vectors pcDNA3.1 and pEGFP-N2 to generate recombinant plasmids HSP60-Flag and HSP60-GFP. The truncations of HSP60 (1–214 aa, 158–432 aa, and 433–573 aa) were cloned into pCMV-HA vector to generate recombinant plasmids HA-1–214 aa, HA-158–432 aa, and HA-433–573 aa. The coding gene of BAX (GenBank no. XM_003127290.5) was cloned into vectors pCMV-HA and pCMV-Myc to generate recombinant plasmids pCMV-HA-BAX and pCMV-Myc-BAX. All constructs were validated by sequencing. The primers used for the construction of recombinant plasmids are listed in Table S2.

### Cell transfection and cell viability analysis

Cells grown to 40–95% confluence were transfected with the specified plasmids in the relevant figures using Lipofectamine LTX reagent or Lipofectamine RNAiMAX reagent according to the manufacturer’s instructions. The cell viability of WSL and WSL-ΔBAX cells was measured by the CCK-8 kit according to the manufacturer’s instructions.

### Annexin V/PI assay, flow cytometry, and TUNEL assay

WSL grown to ~90% confluence was mock transfected or transfected with plasmid MGF360-16R-Myc or pcDNA3.1 for 36 h. The cells were double stained with 10 µL of Annexin V-FITC/PI reagent each for 30 min at room temperature. A total of 20,000 cells were analyzed by flow cytometry using a Flow Cytometer (BD Biosciences, San Jose, CA, USA). In addition, WSL transfected with the plasmids specified in the corresponding figures were fixed with 3.7% paraformaldehyde and permeabilized with 0.2% Triton X-100. The cells were probed with the relevant primary antibodies and then incubated with the corresponding secondary antibodies. After counterstaining cell nuclei with DAPI, the cells were stained with TUNEL reagent for 2 h at room temperature, and then observed by a Nikon Ti2-U fluorescence microscope.

### Confocal immunofluorescence assay

PAMs and WSL grown to ~50% confluence were infected with ASFV-HN09 at an MOI of 2 for the specified times. WSL grown to ~50% confluence was transfected with the specified plasmids for 24 h, and then stained with MitoTracker Red CMXRos 568/647 for 30 min at 37°C before fixation. After fixation with 3.7% paraformaldehyde, the cells were probed with the relevant primary antibodies and then incubated with the corresponding secondary antibodies as described previously (41). After counterstaining cell nuclei with DAPI, the cells were visualized using a Nikon A1 confocal microscope (Nikon Instruments Inc., Tokyo, Japan).

### Immunoblotting, Co-IP, and mass spectrometric identification

PAMs and WSL were infected with ASFV-HN09 at an MOI of 0.1. At the specified times, the cells were harvested by centrifugation and then lysed with Western & IP cell lysis buffer containing 1 mg/mL of protease inhibitor cocktail for 30 min on ice. After centrifugation, the supernatants were analyzed by immunoblotting as described previously (41). WSL was transfected with the specified plasmids for 36 h and then lysed in RIPA lysis buffer containing protease inhibitor on ice for 30 min. After centrifugation, the supernatants were incubated with Anti-Myc, Anti-HA, or Anti-Flag magnetic beads overnight at 4°C with gentle rotation for Co-IP assays. The formed immune complexes bound to the beads were separated by SDS-PAGE. After silver staining, the differentially expressed protein bands displayed on the gel were excised and sent to Shanghai Bioprofile for mass spectrometric identification.

### Mitochondrial isolation and purification

WSL mock transfected or transfected with the plasmids specified in the corresponding figures and ASFV-infected PAMs were subjected to isolation and purification of mitochondria using a commercial mitochondria isolation kit according to the manufacturer’s instructions.

### Construction and identification of MGF360-16R-knockout ASFV

Homologous recombination technology combined with the CRISPR/Cas9 system was used to construct the MGF360-16R-knockout ASFV mutant as previously described (42, 43). Briefly, the donor vector was constructed to comprise a cassette containing the EGFP reporter under the control of the ASFV p72 promoter flanked by a ~1,000 bp-left homologous arm and a ~1,000 bp-right homologous arm (Fig. S5A). PAMs were first infected with ASFV strain HN09 (MOI = 0.5) for 1 h and then transfected with donor vector and sgRNA targeting MGF360-16R (Table S3) using X-tremeGENE HP DNA transfection agent following the manufacturer’s instruction. The cells were incubated at 37°C under 5% CO_2_ for 24 h and observed under a fluorescent microscope. After five rounds of limited dilution combined with fluorescent plaque purification on monolayers of primary PAMs as previously described (43), a monoclonal MGF360-16R-knockout ASFV mutant (designated ∆16R) was successfully obtained and identified by PCR (Fig. S5B; Table S3). Both parental ASFV-HN09 and ∆16R genomes were sequenced using the next-generation sequencing (NGS) technology as previously described (44). Briefly, the DNA of purified ASFV Δ16R and ASFV-HN09 strains was extracted using the QIAamp DNA Mini Kit (51304, Qiagen, Germany), and the viral DNA was purified with the AMPure XP Purification Kit (A63880, Beckman Coulter, USA). The quality and concentration of the DNA were evaluated using the Nanodrop Lite (ND-LITE-PR, ThermoFisher, USA) and the Qubit 4.0 Fluorometer (Q33238, Invitrogen, USA). All procedures were performed according to the manufacturer’s instructions. A standard library was successfully constructed for NGS sequencing using the MGI platform (MGIseq2000). High-quality reads (> Q30) of ASFV were obtained, totaling approximately 8,737,058 reads, with an average sequencing depth of up to 20,000× for the ASFV genome.

### Statistical analysis

All the data were expressed as means ± standard deviation (SD) from three independent experiments. Statistical significance between different groups was analyzed by two-tailed unpaired Student’s *t*-test using GraphPad Prism software version 8.0 (La Jolla, CA, USA). Differences were considered statistically significant at *P < 0.05, **P < 0.01, ***P < 0.001, and ns *P* > 0.05.

## Data Availability

All the data are available upon request.

## SUPPLEMENTAL MATERIAL

Supplemental material jvi.01401-24-s0001.docx Figures S1 to S6; Tables S1 to S3.

ASM does not own the copyrights to Supplemental Material that may be linked to, or accessed through, an article. The authors have granted ASM a non-exclusive, world-wide license to publish the Supplemental Material files. Please contact the corresponding author directly for reuse.
