# Supplementary material for: The African swine fever virus MGF360-16R protein functions as a mitochondrial-dependent apoptosis inducer by competing with BAX to bind to the HSP60 protein
Source: J Virol. 2025 Feb 27;99(4):e01401-24. doi: 10.1128/jvi.01401-24 (PMC11998486; doi:10.1128/jvi.01401-24)
Supplement: Supplemental material — Figures S1 to S6; Tables S1 to S3. [file jvi.01401-24-s0001.docx]

**
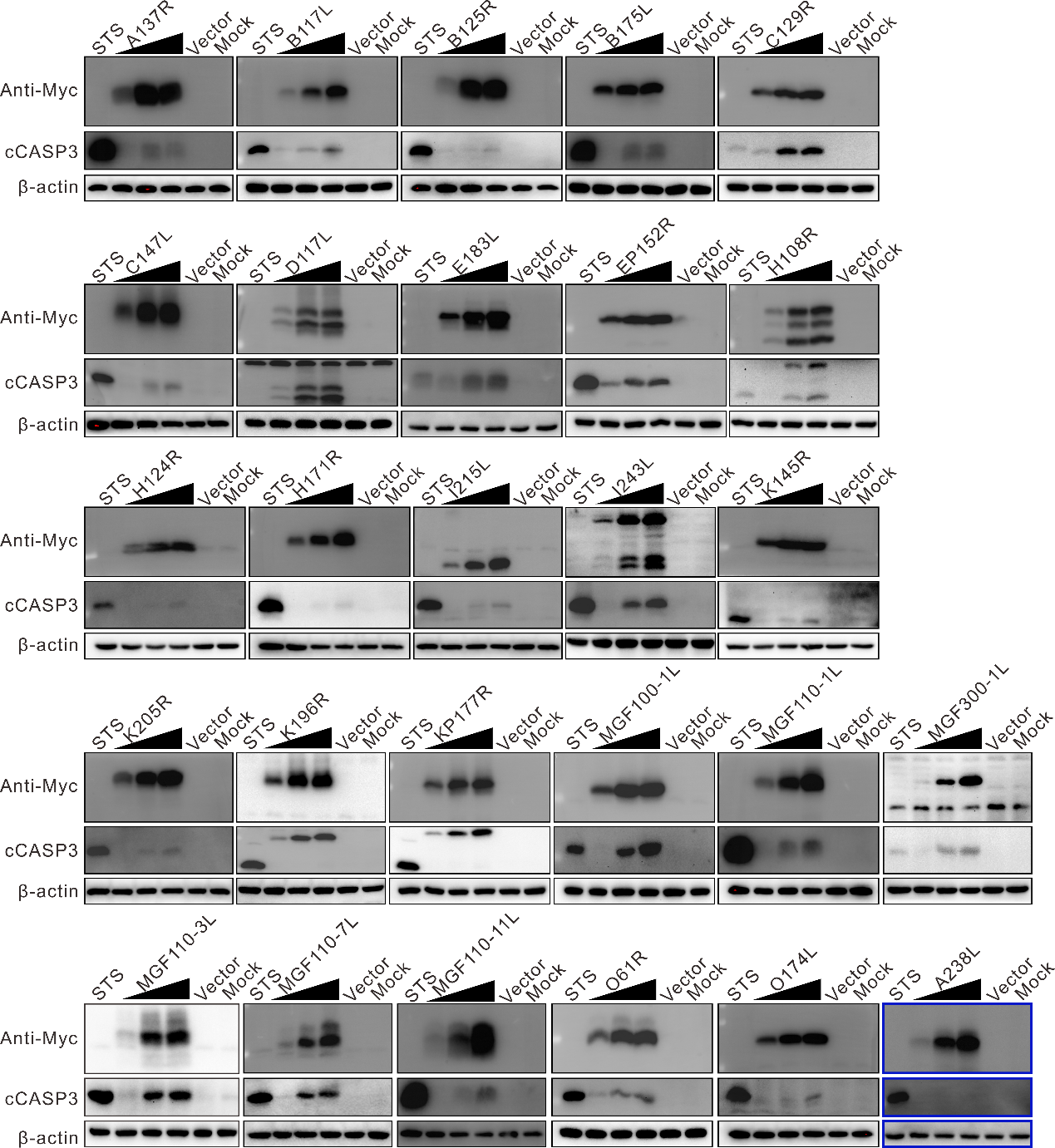
**

**FIG S1** Immunoblotting results of the screened ASFV protein capable of activating caspase-3 (CASP3). WSL was transfected with either an empty vector (pcDNA3.1) or 1, 2, and 3 μg of recombinant plasmids expressing Myc-tagged ASFV proteins A137R, B117L, B125R, B175L, C129R, C147L, D117L, E183L, EP152R, H108R, H124R, H171R, I215L, I243L, K145R, K205R, K196R, KP177R, MGF100-1L, MGF110-1L, MGF300-1L, MGF110-3L, MGF110-7L, MGF110-11L, O61R, O174L, and A238L, respectively. Cells were harvested after 42 h for immunoblotting using antibodies against Myc, CASP3, and β-actin. Cells treated with 2 µM of STS for 4 h were used as a positive control for apoptosis activation. The immunoblotting results of A238L protein, one of the representative proteins judged as negative, was not found to activate CASP3 through immunoblotting screening, are marked with blue boxes. Representative results from experiments performed in three independent biological replicates are shown.


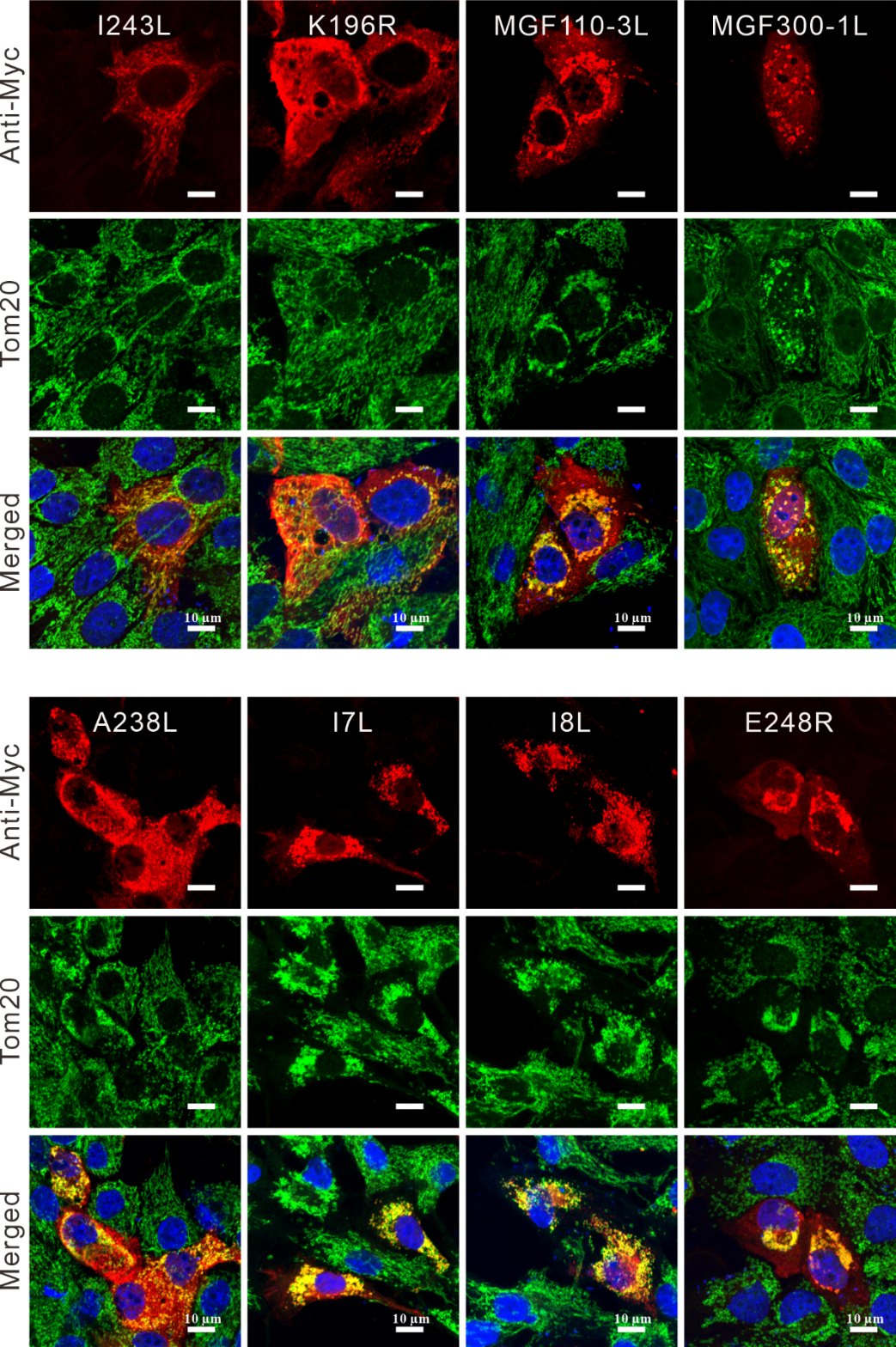


**FIG S2** Confocal immunofluorescence results of selected ASFV proteins that could co-localize with mitochondria. WSL was transfected with either pcDNA3.1 or recombinant plasmids expressing Myc-tagged I243L, K196R, MGF110-3L, MGF300-1L, A238L, I7L, I8L, and E248R proteins, respectively, for 24 h. The cells were then analyzed by confocal immunofluorescence using mouse anti-Myc mAb and rabbit anti-Tom20 pAb as the primary antibodies, followed by immunostaining with Alexa Fluor 488-conjugated goat anti-rabbit and Alexa Fluor 568-conjugated goat anti-mouse IgG secondary antibodies.


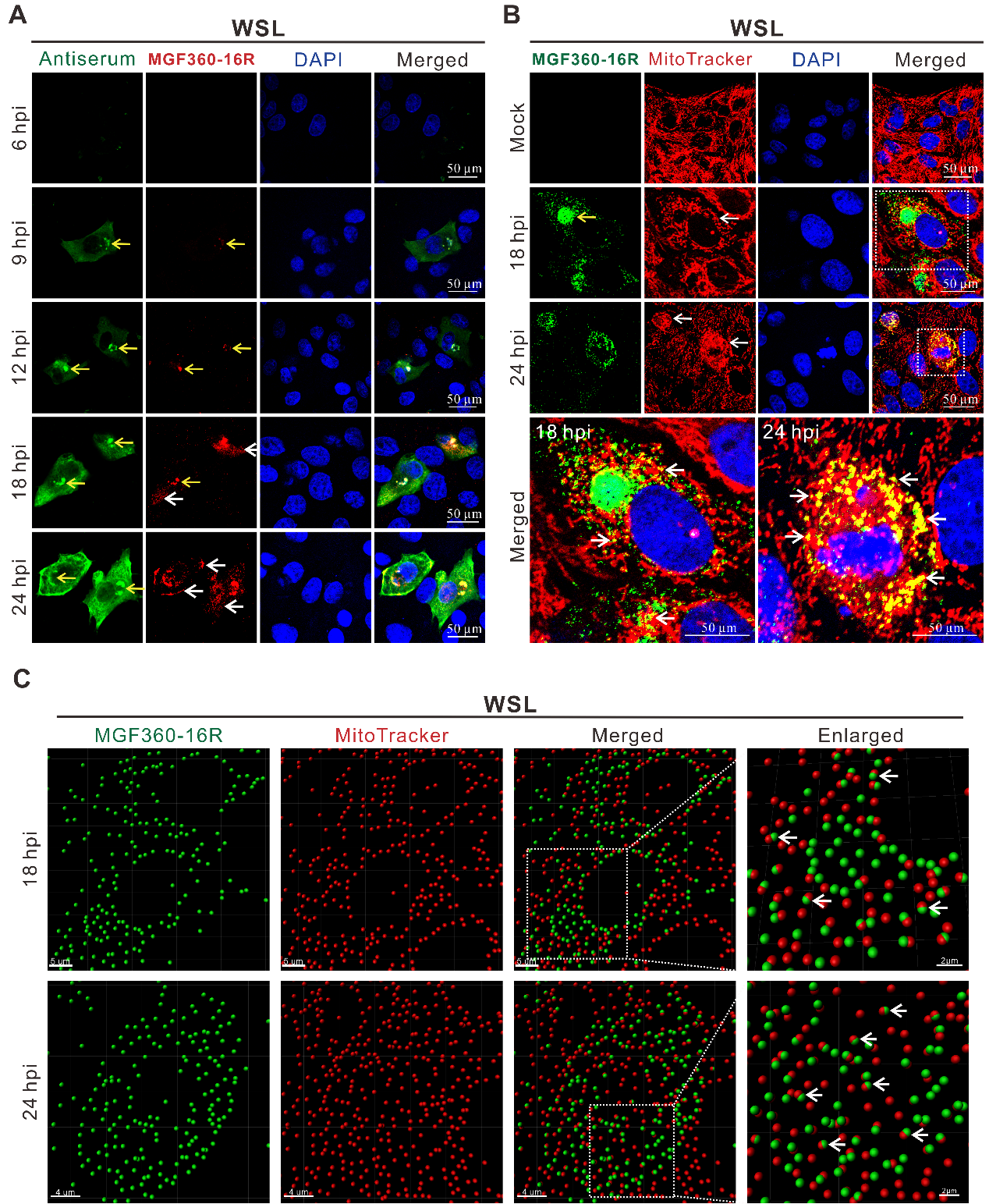


**FIG S3** MGF360-16R protein is localized to viral factory early in infection and partially translocated to mitochondria later in infection. (A) WSL was mock infected or infected with ASFV-HN09 at an MOI of 2. At the indicated times, WSL was fixed and incubated with mouse anti-MGF360-16R pAb and swine ASFV antiserum, followed by immunostaining with Alexa Fluor 568-conjugated goat anti-mouse IgG and FITC-conjugated goat anti-swine IgG, respectively. After counterstaining cell nuclei with DAPI, the cells were observed by a confocal microscope. The representative localization of MGF360-16R protein inside and outside the viral factory is indicated by yellow and white arrows, respectively. (B) WSL was infected as described in (A). At 18 and 24 hpi, WSL was stained with MitoTracker Red CMXRos 647 for 30 min at 37 °C before fixation. Then the cells were incubated with mouse anti-MGF360-16R pAb, followed by immunostaining with Alexa Fluor 488-conjugated goat anti-mouse IgG. After counterstaining cell nuclei with DAPI, the cells were observed by a confocal microscope. The representative localization of MGF360-16R protein inside and outside the viral factory is indicated by yellow and white arrows, respectively. The enlarged image of the area within the white-dashed box is provided below. (C) Three-dimensional reconstruction of co-localization between MGF360-16R protein and mitochondria. The areas within the white-dashed boxes shown in (B) were reconstructed by Imaris software.

**
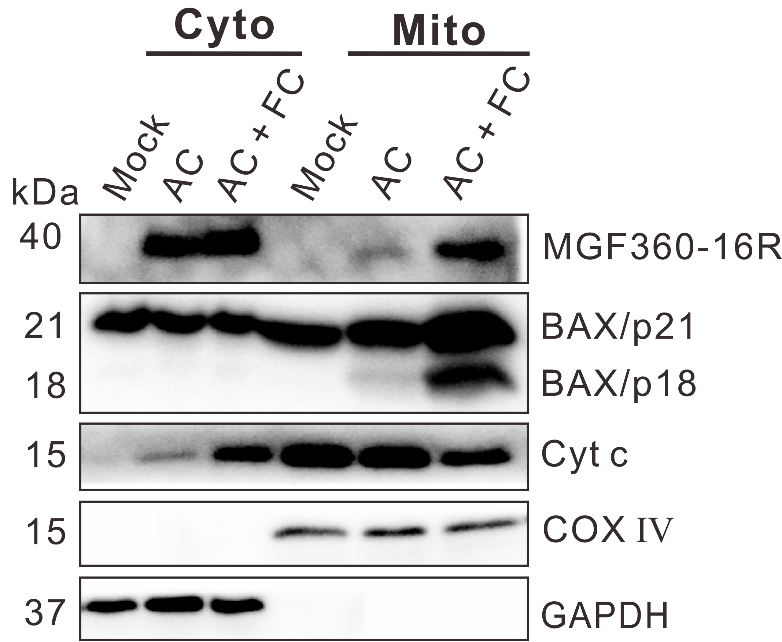
**

**FIG S4** A portion of MGF360-16R protein is transferred to mitochondria in the late stage of ASFV infection. PAMs were mock infected or infected with ASFV-HN09 at an MOI of 2 for 24. Both adherent cells (AC) and floating cells (FC) were harvested by centrifugation. The cytosolic (Cyto) and mitochondrial (Mito) fractions of the cell pellets were separated and detected by immunoblotting using antibodies against MGF360-16R, BAX, cytochrome c (Cyt c), COX IV, and GAPDH. Representative results from experiments performed in three independent biological replicates are shown.


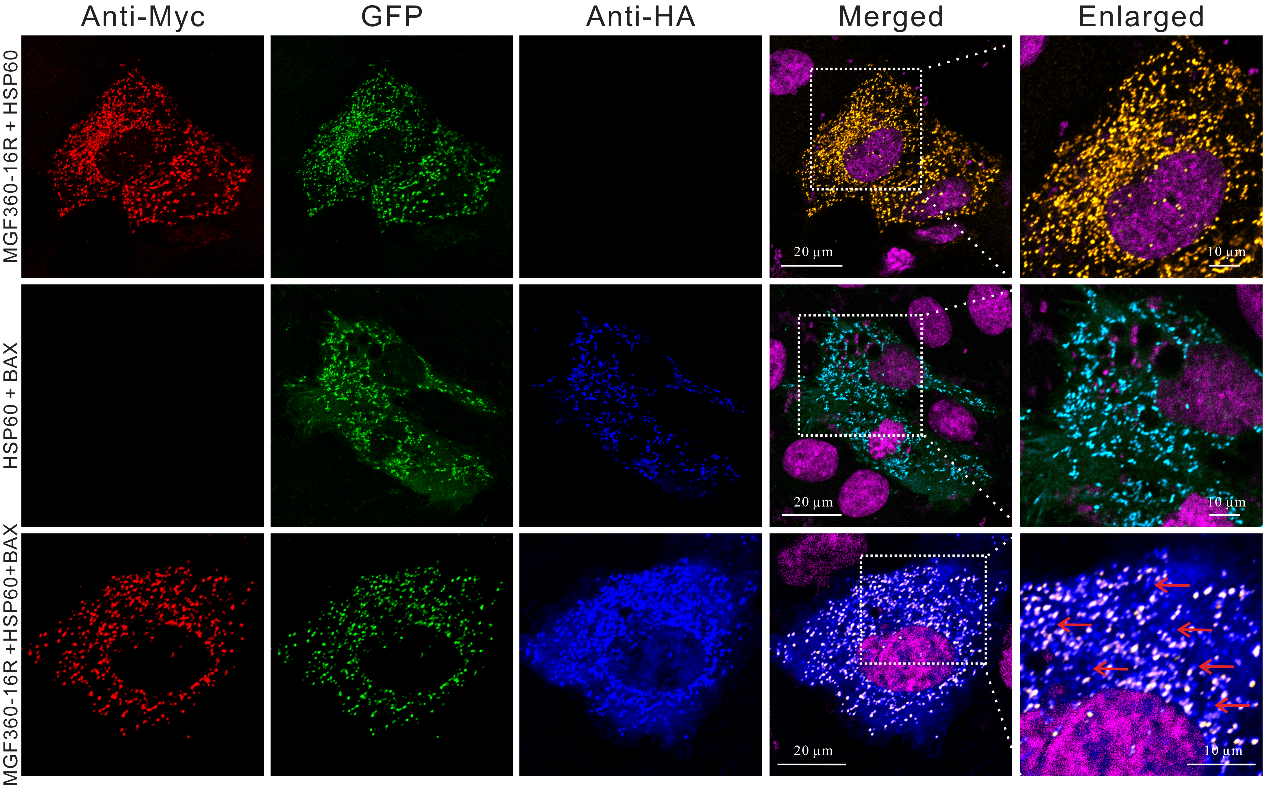


**FIG S5** MGF360-16R protein competes with BAX to bind to HSP60. WSL was double- or triple-transfected with recombinant plasmids expressing Myc-tagged MGF360-16R, GFP-tagged HSP60 or HA-tagged BAX for 24. The cells were fixed and incubated with rabbit anti-Myc and mouse anti-HA antibodies, followed by immunostaining with Alexa Fluor 568-conjugated goat anti-rabbit IgG and Alexa Fluor 647-conjugated goat anti-mouse IgG, respectively. The enlarged image of the area within the white-dashed box is provided on the right.


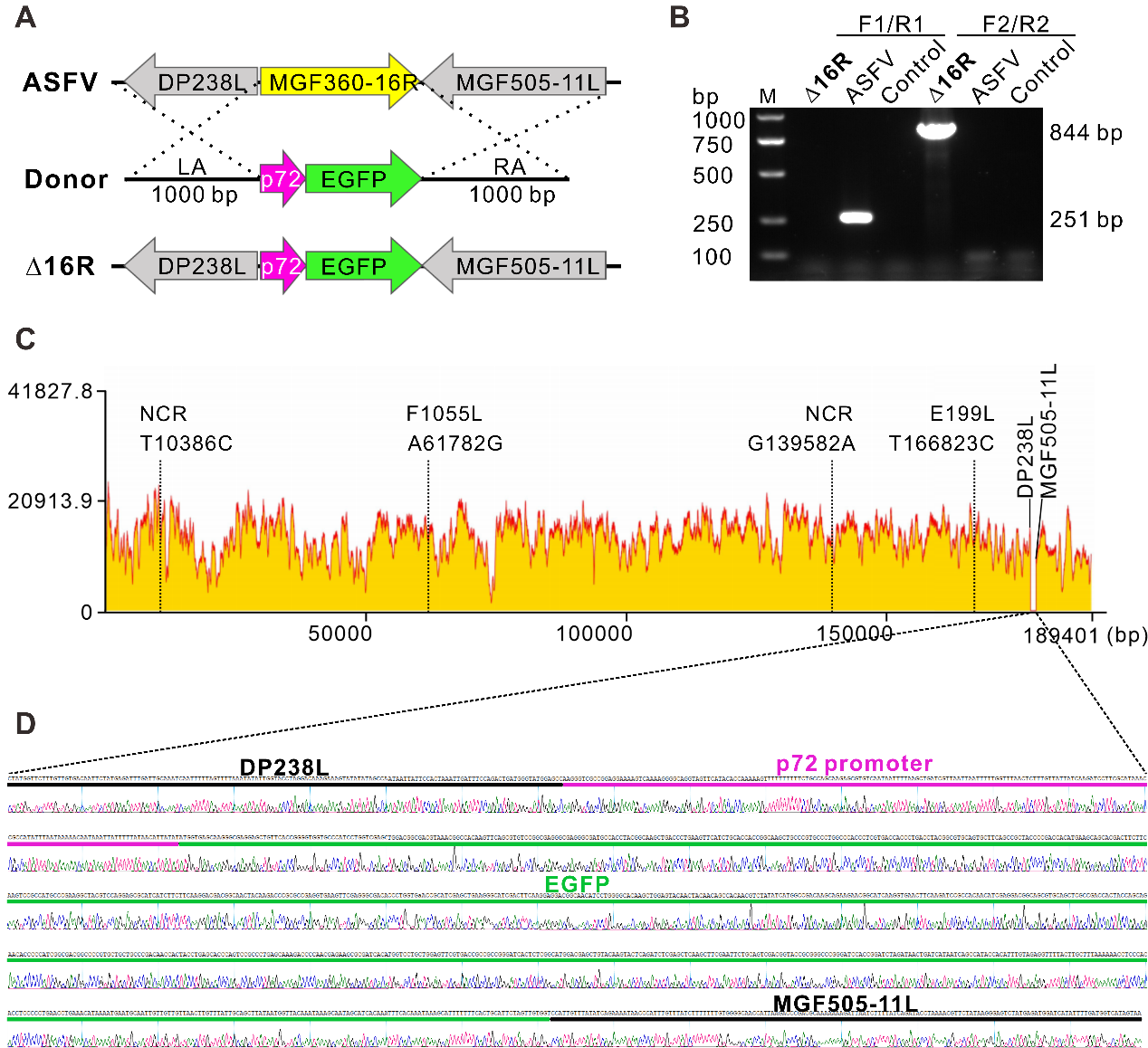


**FIG S6** Construction and identification of MGF360-16R-knockout ASFV mutant. (A) The strategy diagram for constructing MGF360-16R gene-knockout ASFV using homologous recombination technology combined with CRISPR/Cas9 system. The CRISPR/Cas9 system was used to delete the ORF of the MGF360-16R gene (indicated by a yellow arrow) from the parental ASFV-HN09 strain. The donor vector was constructed to comprise a cassette containing the EGFP reporter (indicated by a green arrow) under the control of the ASFV p72 promoter (indicated by a purple arrow) flanked by a ~1000 bp-left homologous (LA) arm and a ~1000 bp-right homologous arm (RA). (B) PCR identification of the purified ASFV ∆16R strain and parental HN09 strain using primer pairs F1/R1 and F2/R2 shown in Table S3. (C) Next-generation sequencing (NGS) analysis of the parental ASFV-HN09 strain and the generated MGF360-16R-knockout ∆16R strain. The complete genome of ASFV ∆16R was sequenced using the parental ASFV as a reference sequence. Compared with the parental strain ASFV-HN09, the genome of ∆16R contains four base mutations (T10386C, A61782G, G139582A, and T166823C) in addition to the cassette introduced to replace MGF360-16R between DP238L and MGF505-11L genes, two of which are located in the non-coding region (NCR), and the other two are located in the F1055L and E199L genes, respectively. The Y-axis represents the depth of sequencing of the NGS technology, while the X-axis represents nucleotide position (bp). Mutations marked by dotted lines. (D) Detailed information and specific location of the inserted cassette containing an EGFP reporter under the control of the ASFV p72 promoter used for replacing the MGF360-16R gene.

**TABLE S1** Information of the178 ASFV proteins used for screening proteins capable of inducing apoptosis

|  | **ASFV proteins** | **ASFV proteins capable of activating caspase-3 screened by western blot** | **ASFV proteins co-localized with mitochondria screened by confocal immunofluorescence** | **ASFV proteins that can both activate caspase-3 and co-localize with mitochondria** |
| --- | --- | --- | --- | --- |
|  | A104R, A118R, A137R, A151R, A179L, A224L, A238L, A240L, A859R, B66L, B117L, B119L, B125R, B169L, B175L, B263R, B318L, B354L, B385R, B407L, B438L, B475L, B602L, B646L, B962L, C62L, C84L, C122R, C129R, C147L, C257L, C315R, C475L, C717R, C962R, CP80R, CP123L, CP204L, CP312R, CP530R, CP2475L, D117L, D129L, D205R, D250R, D339L, D345L, D1133L, DP60R, DP71L, DP79L, DP96R, DP238L, E66L, E111L, E120R, E146L, E165R, E183L, E184L, E199L, E248R, E301R, E423R, EP84R, EP152R, EP153R, EP296R, EP364R, EP402R, EP424R, EP1242L, F165R, F317L, F334L, F778R, G1211R, G1340L, H108R, H124R, H171R, H233R, H240R, H339R, H359L, I7L, I8L, I9R, I10L, I73R, I177L, I196L, I215L, I226R, I243L, I267L, I329L, K78R, K145R, K196R, K205R, K421R, KP177R, L11L, L60L, L83L, M448R, M1429L, MGF100-1L, MGF100-1R, MGF110-1L, MGF110-2L, MGF110-3L, MGF110-4L, MGF110-5L-6L, MGF110-7L, MGF110-9L, MGF110-11L, MGF110-12L, MGF110-13L, MGF110-14L, MGF300-1L, MGF300-4L, MGF360-1L, MGF360-2L, MGF360-3L, MGF360-4L, MGF360-6L, MGF360-8L, MGF360-9L, MGF360-10L, MGF360-11L, MGF360-11R, MGF360-12L, MGF360-13L, MGF360-14L, MGF360-15R, MGF360-16R, MGF360-18R, MGF360-21R, MGF505-1R, MGF505-2R, MGF505-3R, MGF505-4R, MGF505-5R, MGF505-6R, MGF505-9R, MGF505-10R, MGF505-11L, NP419L, NP868R, NP1450L, O61R, 0174L, P1192R, Q706L, QP383R, QP509L, R298L, S183L, S273R, X69R, ASFV_G_ACD_00090, ASFV_G_ACD_00120, ASFV_G_ACD_00160, ASFV_G_ACD_00190, ASFV_G_ACD_00210, ASFV_G_ACD_00240, ASFV_G_ACD_00270, ASFV_G_ACD_00290, ASFV_G_ACD_00320, ASFV_G_ACD_00330, ASFV_G_ACD_00350, ASFV_G_ACD_00360, ASFV_G_ACD_01020, ASFV_G_ACD_01760, ASFV_G_ACD_01940, ASFV_G_ACD_01960 | A137R, B117L, B125R, B175L, C129R, C147L, D117L, E183L, EP152R, H108R, H124R, H171R, I215L, I243L, K196R, K145R, K205R, KP177R, MGF100-1L, MGF110-1L, MGF110-3L, MGF110-7L, MGF110-11L, MGF300-1L, MGF360-16R, O61R, and O174L | A238L, E248R, I7L, I8L, I243L, K196R, MGF110-3L, MGF300-1L, and MGF360-16R | K196R, I243L, MGF110-3L, MGF300-1L, and MGF360-16R |
| In total | 178 | 27 | 9 | 5 |

**TABLE S2** The primers used for constructing recombinant plasmids

| **Primers** | **Sequences (5’-3’)** | **Restriction enzymes** |
| --- | --- | --- |
| 1-145aa-Myc-F | CTTGGTACCGAGCTCGGATCCACATGCTGAGCCTGCAGACCATCG | *BamH Ⅰ* |
| 1-145aa-Myc-R | TCTAGACTCGAGCGGCCGCTTACAGATCCTCTTCAGAGATGAGTTT  CTGCTCGTCGTCGAAGATCTCGTAGC | *Not Ⅰ* |
| 40-215aa-Myc-F | CTTGGTACCGAGCTCGGATCCACATGCCCATCAAGATCTGCAA | *BamH Ⅰ* |
| 40-215aa-Myc-R | TCTAGACTCGAGCGGCCGCTTACAGATCCTCTTCAGAGATGAGTTT  CTGCTCGCGCAGGGAGAAGGGCTTGA | *Not Ⅰ* |
| 146-352aa-Myc-F | CTTGGTACCGAGCTCGGATCCACATGAACAGCGTGCTGGACTGT | *BamH Ⅰ* |
| 146-352aa-Myc-R | TCTAGACTCGAGCGGCCGCTTACAGATCCTCTTCAGAGATGAGTTT  CTGCTCCTGGCAGTAGTAGGTGAACACG | *Not Ⅰ* |
| HSP60-Flag-F | GTACCGAGCTCGGATCCACATGCTTCGATTACCCGCAGTC | *BamH Ⅰ* |
| HSP60-Flag-R | TCTAGACTCGAGCGGCCGCTTATTTGTCGTCGTCGTCCTTGTAATC  GAACATGCCACCTCCCATAC | *Not Ⅰ* |
| HSP60-GFP-F | TCAAGCTTCGAATTCATGCTTCGATTACCCGCAGTC | *EcoR Ⅰ* |
| HSP60-GFP-R | CGGCCGGTGGATCCCTTTGTCGTCGTCGTCCTTGTAATC | *BamH Ⅰ* |
| HA-1–214aa-F | TGGCCATGGAGGCCCGAATTCGGCTTCGATTACCCGCAGTC | *EcoR Ⅰ* |
| HA-1–214aa-R | GCGGCCGCGGTACCTCGAGATTATATAATCTCTAATTCATCAT | *Xho Ⅰ* |
| HA-158–432aa-F | TGGCCATGGAGGCCCGAATTCGGCAGTCTAAACCTGTGACAAC | *EcoR Ⅰ* |
| HA-158–432aa-R | GCGGCCGCGGTACCTCGAGATTAAACAGCAGCTCTTGTCGCAT | *Xho Ⅰ* |
| HA-433–573aa-F | TGGCCATGGAGGCCCGAATTCGGGAAGAAGGCATCGTTC | *EcoR Ⅰ* |
| HA-433–573aa-R | GCGGCCGCGGTACCTCGAGATTAGAACATGCCACCTCCCATAC | *Xho Ⅰ* |
| pCMV-Myc-BAX-F | GCCATGGAGGCCCGAATTCGGGACGGGTCCGGGGAG | *EcoR Ⅰ* |
| pCMV-Myc-BAX-R | CGGCCGCGGTACCTCGAGATTAGCCCATCTTCTTCCAGA | *Xho Ⅰ* |
| pCMV-HA-BAX-F | TGGCCATGGAGGCCCGAATTCGGGACGGGTCCGGGGAGCA | *EcoR Ⅰ* |
| pCMV-HA-BAX-R | GCGGCCGCGGTACCTCGAGATTAGCCCATCTTCTTCCAGATG | *Xho Ⅰ* |

F: forward primer; R: reverse primer. Restriction sites are underlined.

**TABLE S3** The primers used for the construction and identification of *MGF360-16R*-knockout ASFV

| **Name** | **Sequence（5′-3′）** |
| --- | --- |
| sgRNA (9)-sense-F | CACCGTCTGCAGACGATCGCAAAAA |
| sgRNA (9)-sense-R | AAACTTTTTGCGATCGTCTGCAGAC |
| sgRNA (489)-sense-F | CACCGAAGGCCCGTATACCTCTCA |
| sgRNA (489)-sense-R | AAACTGAGAGGTATACGGGCCTTC |
| Left-arm-F | CTCGAGGTCGACGGTATCGATAAGCTTGACTGTCGTTAAATAATTACTACCTATT |
| Left-arm-R | GGCTCCATACCCATCAGTCTGGAAAT |
| p72-promoter-F | TTTTCTATACAGGGGGTCGCCGGAGGAAAAG |
| p72-promoter-R | CGCCCTTGCTCACCATTATATAATGTTATAAAAATAATTTATTGTTTTTATTAAA |
| EGFP-F | ATTTCCAGACTGATGGGTATGGAGCCAAGGGTCGCCGGAGGAAAAGTCAAA |
| EGFP-R | AATGGGTTATTTTCTGATATAAACAATGGACCACAACTAGAATGCAGTGAA |
| Right-arm-F | CCATTGTTTATATCAGAAAATAACCCATT |
| Right-arm-R | CGGCCGCTCTAGAACTAGTGGATCCCACAGCGATCCAAAAAAATTACTTCCGT |
| F1 | TATAGCCAATAATTATTCCACTAAATTGA |
| R1 | TCCAGACTCATTCCATGATTG |
| F2 | TATAGCCAATAATTATTCCACTAAATTGA |
| R2 | ACTGGGTGCTCAGGTAGTGGTTGTCGGG |
| F: forward primer; R: reverse primer. | |
